# Image-Guided Nanodelivery of Pt(IV) Prodrugs to GRP-Receptor Positive Tumors

**DOI:** 10.7150/ntno.78807

**Published:** 2023-01-01

**Authors:** Francisco Silva, Carolina Mendes, Alice D'Onofrio, Maria Paula Cabral Campello, Fernanda Marques, Teresa Pinheiro, Kyle Gonçalves, Sérgio Figueiredo, Lurdes Gano, Mauro Ravera, Elisabetta Gabano, António Paulo

**Affiliations:** 1 Centro de Ciências e Tecnologias Nucleares, Instituto Superior Técnico, Universidade de Lisboa, Campus Tecnológico e Nuclear, Estrada Nacional 10, Km 139.7, 2695-066 Bobadela LRS, Portugal.; 2 Departamento de Engenharia e Ciências Nucleares, Instituto Superior Técnico, Universidade de Lisboa, Portugal.; 3 iBB - Instituto de Bioengenharia e Biociências, Instituto Superior Técnico, Universidade de Lisboa, Av. Rovisco Pais 1, 1049-001 Lisboa, Portugal.; 4 Institute for Systems and Robotics (ISR), LARSyS, Instituto Superior Técnico, Department of Bioengineering, Universidade de Lisboa, Portugal; 5 H&TRC - Health &Technology Research Center, ESTeSL/IPL - Escola Superior de Tecnologia da Saúde de Lisboa/Instituto Politécnico de Lisboa, Portugal.; 6 Dipartimento di Scienze e Innovazione Tecnologica, Università del Piemonte Orientale, Viale Teresa Michel 11, 15121 Alessandria, Italy.; 7 Dipartimento per lo Sviluppo Sostenibile e la Transizione Ecologica, Università del Piemonte Orientale, Piazza Sant'Eusebio 5, 13100 Vercelli, Italy.

**Keywords:** gold nanoparticles, bombesin peptides, Pt(IV) prodrugs, ^67^Ga, prostate cancer

## Abstract

Over the last decades, gold nanoparticles (AuNPs) have proven to be remarkable tools for drug delivery and theranostic applications in cancer treatment. On the other hand, Pt(IV) prodrugs have been employed as an interesting alternative to the more common Pt(II) complexes, such as cisplatin, for cancer chemotherapy. Searching to design an image-guided nanocarrier to deliver selectively Pt(IV) prodrugs to tumors expressing the gastrin releasing peptide receptor (GRPR), we have synthesized small core AuNPs carrying a thiolated DOTA derivative, a GRPR-targeting bombesin analog (BBN[7-14]) and a Pt(IV) prodrug attached to the AuNPs without (**AuNP-BBN-Pt1**) or with a PEGylated linker (**AuNP-BBN-Pt2** and **AuNP-BBN-Pt3**). In the GRPR+ prostate cancer PC3 cell line, the cytotoxic activity of the designed **AuNP-BBN-Pt** nanoparticles is strongly influenced by the presence of the PEGylated linker. Thus, **AuNP-BBN-Pt1** displayed the lowest IC_50_ value (9.3 ± 2.3 µM of Pt), which is comparable to that exhibited by cisplatin in the same cell line. In contrast, **AuNP-BBN-Pt1** showed an IC_50_ value of 97 ± 18 µM of Pt in the non-tumoral RWPE-1 prostate cells with a much higher selective index (SI) towards PC3 cells (SI = 10) when compared with cisplatin (SI = 1.3). The AuNPs were also successfully labeled with ^67^Ga and the resulting **^67^Ga-AuNP-BBN-Pt** were used to assess their cellular uptake in PC3 cells, with **AuNP-BBN-Pt1** also displaying the highest cellular internalization. Finally, intratumoral administration of **^67^Ga-AuNP-BBN-Pt1** in a PC3 tumor-bearing mice showed a prolonged retention of the nanoparticle compared to that of cisplatin, with optimal *in vivo* stability and 20% of the injected platinum remaining in the tumor after 72 h post-injection. Furthermore, microSPECT imaging studies confirmed the uptake and considerable retention of the ^67^Ga-labeled AuNPs in the tumors. Overall, these results show the potential of these targeted AuNPs loaded with Pt(IV) prodrugs for prostate cancer theranostics.

## Introduction

Metal nanoparticles (NPs) have been sparking interest in biomedical research because of their favorable features for diagnostic and therapeutic applications. These include their availability in a varied range of sizes and shapes and their large surface area to volume ratio that allow versatile chemical modifications with different imaging and therapeutic entities and enable the development of innovative theranostic strategies. The most commonly studied systems include gold, silver, titanium oxide and iron nanoparticles, as well as quantum dots 1-3. Among these different inorganic NPs, gold nanoparticles (AuNPs) have emerged as promising tools for biomedical applications due to their good biocompatibility and low toxicity and immunogenicity 4-6. These favorable characteristics led, in recent years, to several clinical trials with AuNP-based drugs, namely to treat Parkinson's disease, Type 1 diabetes, and also cardiovascular and oncological diseases 7.

For NP-based cancer treatments, the most significant progress has been obtained with organic nanoparticles such as Doxil®, the first FDA-approved nanodrug consisting of PEGylated liposomes loaded with the anticancer agent doxorubicin 8. Nevertheless, human clinical trials for the treatment of solid tumors with AuNPs have also led to encouraging results, namely with Auroimune® (CYT-6091) used for the delivery of recombinant human TNF-α protein as an anticancer therapy in late-stage pancreatic, breast, colon, melanoma, sarcoma and lung cancer patients 9. Consequently, several other AuNPs were studied as nanocarriers for the delivery of cytotoxic drugs, such as paclitaxel, doxorubicin, gemcitabine or Pt-based drugs 4-7. In the latter case, AuNPs have been evaluated in the delivery of cisplatin using different types of approaches aiming to enhance anticancer effects at lower doses and to reduce the most adverse effects caused by cisplatin (nephrotoxicity, neurotoxicity, myelosuppression, and emesis, in particular) in order to obtain a more safe clinical use of this anticancer drug 10-15.

**Scheme 1:**
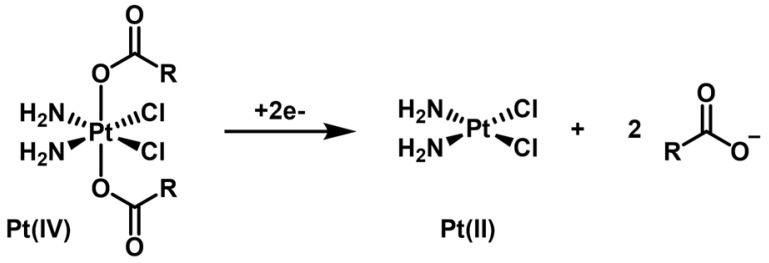
Scheme of the 2e^-^ reduction of a generic cisplatin-based dicarboxylato Pt(IV) complex (*activation by reduction*).

Octahedral Pt(IV) complexes have emerged in the past years as an interesting alternative to the more common square-planar Pt(II) compounds like cisplatin 16, 17. Pt(IV) complexes are relatively inert in the circulation and act as prodrugs that are converted to pharmacologically active inside the cancer cells. In fact, in the hypoxic tumor environment, biological reductants, such as glutathione or ascorbic acid, intracellularly reduce the inactive Pt(IV) complexes to the cytotoxic Pt(II) compounds (*activation by reduction*, Scheme 1). Usually, the Pt(II) complexes contain aliphatic or aromatic amines as non-labile basal ligands and more labile chloridos or carboxylato ligands. Upon reduction of the Pt(IV) center, the aquation/activation of the labile ligands enables platination reactions with the purine bases of DNA, generating Pt(II)-DNA adducts that induce cell death by apoptosis. The Pt(IV) complexes contain two extra ligands, the axial ones, which are lost on the reduction of Pt(IV) to Pt(II). When preparing the Pt(IV) prodrug, the added axial ligands of these Pt(IV) complexes are typically hydroxide or chloride. In particular, axial hydroxido ligands can be carboxylated to attach functional or targeting groups and/or bioactive ligands. This type of approach has also been used to link Pt(IV) complexes to the surface of nanoparticles, including AuNPs, in order to achieve a more selective and/or enhanced internalization of the Pt compounds by tumor cells 16.

The study of the delivery of Pt(IV) prodrugs by AuNPs has been pioneered by Lippard et al., who have engineered AuNPs with thiolated oligonucleotides carrying a terminal dodecylamine and conjugated to a Pt(IV) prodrug through a succinate group coordinated at an axial position. The final construct showed high antiproliferative activity against different human cancer cell lines and was more effective than cisplatin 18. Later on, Liu et al. used a similar approach to functionalize PEGylated gold nanorods with a similar succinate-containing Pt(IV) prodrug. The final functionalized nanorods showed high cellular uptake in different types of cancer cells and superior cytotoxicity when compared to cisplatin, overcoming the chemoresistance often associated with the use of cisplatin 19. Several other studies have been reported for the delivery of Pt(IV) prodrugs by AuNPs, including few rare examples of targeted drug delivery approaches 16, 20-22. For instance, glutathione stabilized AuNPs functionalized with a small peptide (CRGDK) targeting the Neuropilin-1 (Nrp-1) receptor were evaluated *in vitro* as nanoplatforms for the specific delivery of a Pt(IV) drug to prostate cancer cells 23. The Nrp-1 receptor plays a significant role during angiogenesis and is expressed by a wide variety of tumors, namely prostate cancer. The AuNPs targeted at the Nrp-1 showed enhanced cellular uptake and cytotoxicity in cancer cells, when compared with non-targeted counterparts. However, despite the potential advantages of targeted drug delivery over passive drug delivery, targeted approaches based on AuNPs for the delivery of Pt(IV) prodrugs have seldom been studied.

We and others have shown that AuNPs functionalized with bombesin derivatives were able to specifically deliver imaging (e.g., ^67^Ga) and therapeutic radionuclides (e.g., ^198^Au and ^177^Lu) to tumor cells overexpressing the gastrin releasing peptide receptor (GRPR) 24-29. In particular, our group proceeded with the functionalization of spherical and small-sized (ca. 4 nm gold core) AuNPs with a thioctic acid derivative of the BBN [7-14] peptide, which led to target-specific NPs (**AuNP-BBN**) with high affinity for GRPR 28. These AuNPs are stabilized with a thiolated macrocyclic derivative, TDOTA (2-[4,7-bis(carboxymethyl)-10-[2-(3-sulfanylpropanoylamino)ethyl]-1,4,7,10-tetrazacyclododec-1-yl]acetic acid), which provides them with highly negative zeta potential values and avoid aggregation in solution. The presence of the DOTA chelator ensures a stable coordination of a variety of metal ions useful for diagnostic and therapeutic applications, as we have shown for Gd^3+^ and ^67^Ga^3+^ which are of interest for magnetic resonance imaging (MRI) and single photon emission computed tomography (SPECT), respectively 29. Most relevantly, the ^67^Ga-labeled **AuNP-BBN** presented high cellular internalization in human prostate cancer PC3 cells overexpressing the GRPR and a significant tumor uptake in mice bearing prostate cancer xenografts. Furthermore, **AuNP-BBN** performed as selective and effective radiosensitizers for PC3 cells upon their exposure to γ-photon irradiation, reflecting the higher uptake of **AuNP-BBN** in PC3 cells when compared with normal RWPE-1 prostate cells 30.

**Figure 1:**
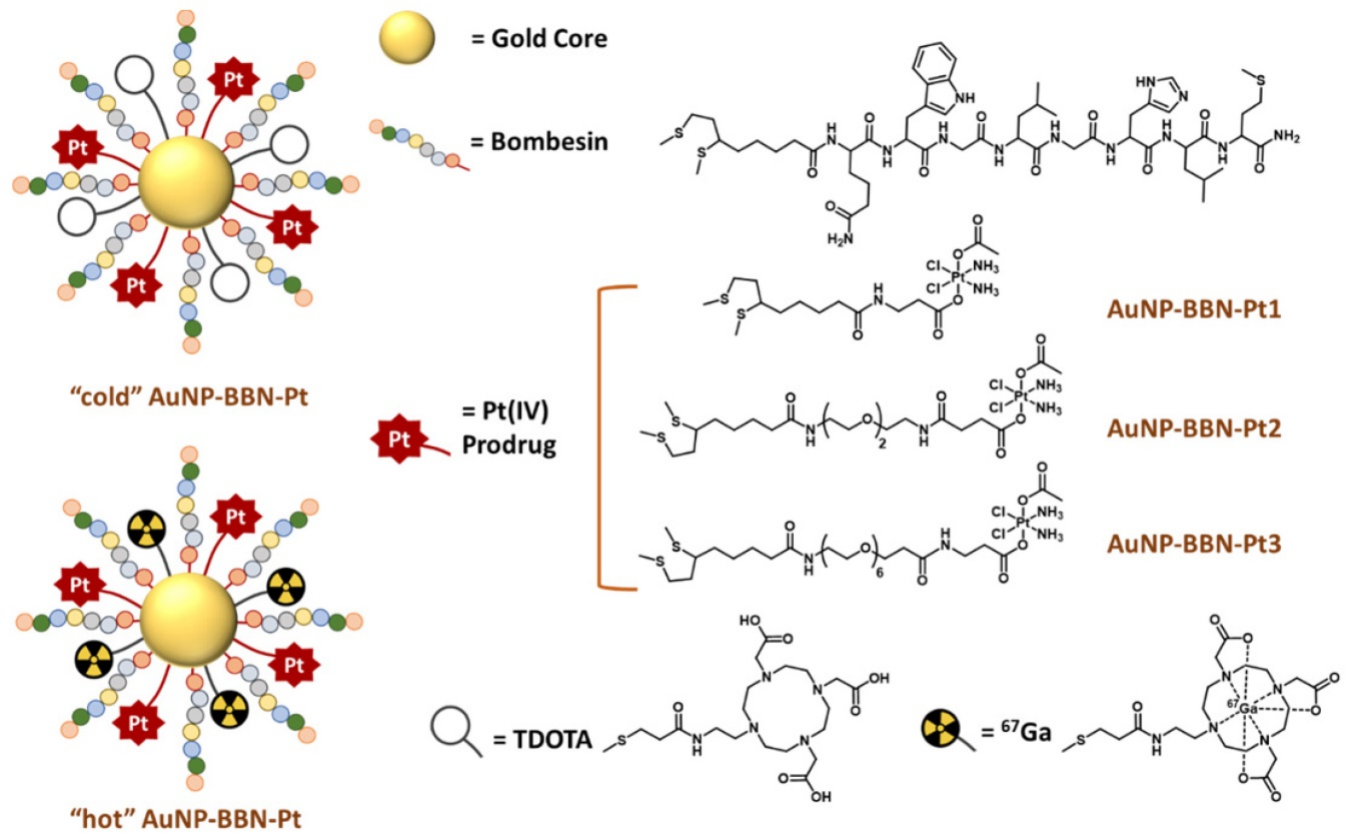
Schematic drawing of the chemical structure of **AuNP-BBN-Pt** nanoparticles.

Altogether, the favorable biological features exhibited by **AuNP-BBN** prompted us to study the suitability of this nanoplatform for the delivery of Pt(IV) prodrugs to GRPR-positive tumors, within an image-guided approach taking advantage of the possibility to label **AuNP-BBN** with a medical imaging radionuclide. For this purpose, we have functionalized **AuNP-BBN** with thioctic acid derivatives of Pt(IV) prodrugs and proceeded with the ^67^Ga-labeling of the resulting Pt-containing NPs (**AuNP-BBN-Pt**, Figure 1**)**. By considering ^67^Ga for the labeling of AuNP-BBN-Pt, we have taken into consideration that ^67^Ga allowed stable radiolabeling of the original nanoplatform **AuNP-BBN** 28, 29. Furthermore, ^67^Ga is a commercially available SPECT radionuclide with a relatively long half-life (T_1/2_ = 3.27 d) that is very well suited to follow *tumor* uptake and retention of the radiolabeled **AuNP-BBN-Pt** *in vivo* 24.

For the present study, we have used (*OC*‑6‑44)‑acetato(β‑alaninato)diamminedichloridoplatinum(IV) (**Pt-Ac-Ala**) and (*OC*-6-44)-acetatodiammine(3-carboxypropanoato)dichloridoplatinum(IV) (**Pt-Ac-Suc**) as asymmetric Pt(IV) prodrugs, which contain: i) the equatorial arrangement of cisplatin; ii) an inert acetate axial ligand; iii) axial ligands (β‑alaninato or 3-carboxypropanoato) (Figure 1). The latter contain terminal amino and carboxylic acid functions suitable for selective coupling to thioctic acid derivatives and further coupling to the AuNPs. Previously, these types of Pt(IV) prodrugs were already loaded into silica nanoparticles (SNPs) for the passive delivery of cisplatin to human tumors, and *in vitro* studies confirmed that cytosol extracted from tumor cells can quickly and efficiently reduce these Pt(IV) prodrugs and produce the active cisplatin drug 31-33.

The clinical relevance of our target-specific nanoplatforms for the delivery of anticancer Pt(IV) prodrugs arises from the overexpression of GRPR in several human cancers not effectively treated with cisplatin and related Pt(II) derivatives such as glioblastoma, prostate, colon, pancreas, breast, small cell lung, non-small cell lung (NSCL) and ovarian cancers 34, 35. To the best of our knowledge, the GRPR-targeted delivery of Pt(IV) prodrugs to human cancer cells has not been investigated so far. However, few examples of GRPR-targeted delivery strategies have been reported for other anticancer drugs (e.g., doxorubicin, paclitaxel, and docetaxel), based mainly on liposomes and polymeric nanoparticles to treat NSCL and prostate cancers 36, 37. When searching for an initial validation of **AuNP-BBN-Pt** as new tools for the image-guided and selective delivery of Pt(IV) prodrugs to tumor cells and tissues, we have focused on prostate cancer (PCa), as this type of cancer is known to overexpress GRPR in a relatively large percentage of cases. To tackle this goal, we have synthesized new thioctic acid derivatives of the **Pt-Ac-Ala** and **Pt-Ac-Suc** prodrugs containing different methylene or polyethylene glycol (PEG) linkers between the Pt(IV) complexes and the thioctic acid unit and studied their linkage to the **AuNP-BBN** nanoplatform.

In this contribution, we report on the detailed physico-chemical characterization of the resulting Pt-containing **AuNP-BBN-Pt** (**Pt = Pt1-Pt3**) nanoparticles (see Figure 1) and describe their preclinical evaluation in the prostate cancer PC3 cell line overexpressing the GRPR. The *in vitro* studies comprised the screening of the cytotoxicity of the Pt-containing AuNPs in this cell line, as well as uptake and internalization studies involving radiometric methods that were performed using the ^67^Ga-labeled counterparts (i.e., **^67^Ga**-**AuNP-BBN-Pt**, where **Pt = Pt1-Pt3**). Furthermore, the biological behavior of a selected target-specific nanoplatform (**AuNP-BBN-Pt1**) was studied in more detail in tumor and non-tumor cells, in comparison with cisplatin. This comparative study was expected to address how the GRPR-targeting strategy would influence the selectivity of cytotoxic effects (i.e., between tumor and non-tumor cells), and the induced mechanisms of cell death, namely through ROS production and cell apoptosis. Lastly, we report on biodistribution and microSPECT-CT imaging studies for **^67^Ga**-**AuNP-BBN-Pt1**, upon intratumoral administration of this radiolabeled nanoconstruct in BALB/c nude mice bearing human prostate PC3 xenografts. Animal studies comprised quantification of the accumulation of ^67^Ga, Au, and Pt in the tumors and in selected organs and tissues, with the aim of confirming the feasibility of image-guided nanodelivery of Pt(IV) prodrugs with the target-specific **AuNP-BBN** nanoplatform.

## Results and Discussion

### Synthesis and characterization of thioctic acid derivatives of Pt(IV) prodrugs

As we have previously done for the functionalization of **AuNP-TDOTA** with the bioactive BBN [7-14] sequence 28, we have thought on thioctic acid derivatives to attach the Pt(IV) prodrugs to the surface of the **AuNP-BBN**. The thioctic acid group spontaneously reacts at room temperature with the Au atoms from the NPs surface, through bidentate coordination to gold that leads to more stable Au-S bonds, under physiological and biological conditions, when compared with unidentate thiols. These advantages prompted other authors to use thioctic acid derivatives to attach organic cytotoxic drugs (e.g., doxorubicin) or even cisplatin-like compounds to AuNP carriers 38, 39. However, to the best of our knowledge, the use of thioctic derivatives to functionalize AuNPs with Pt(IV) prodrugs was unprecedented.

**Scheme 2:**
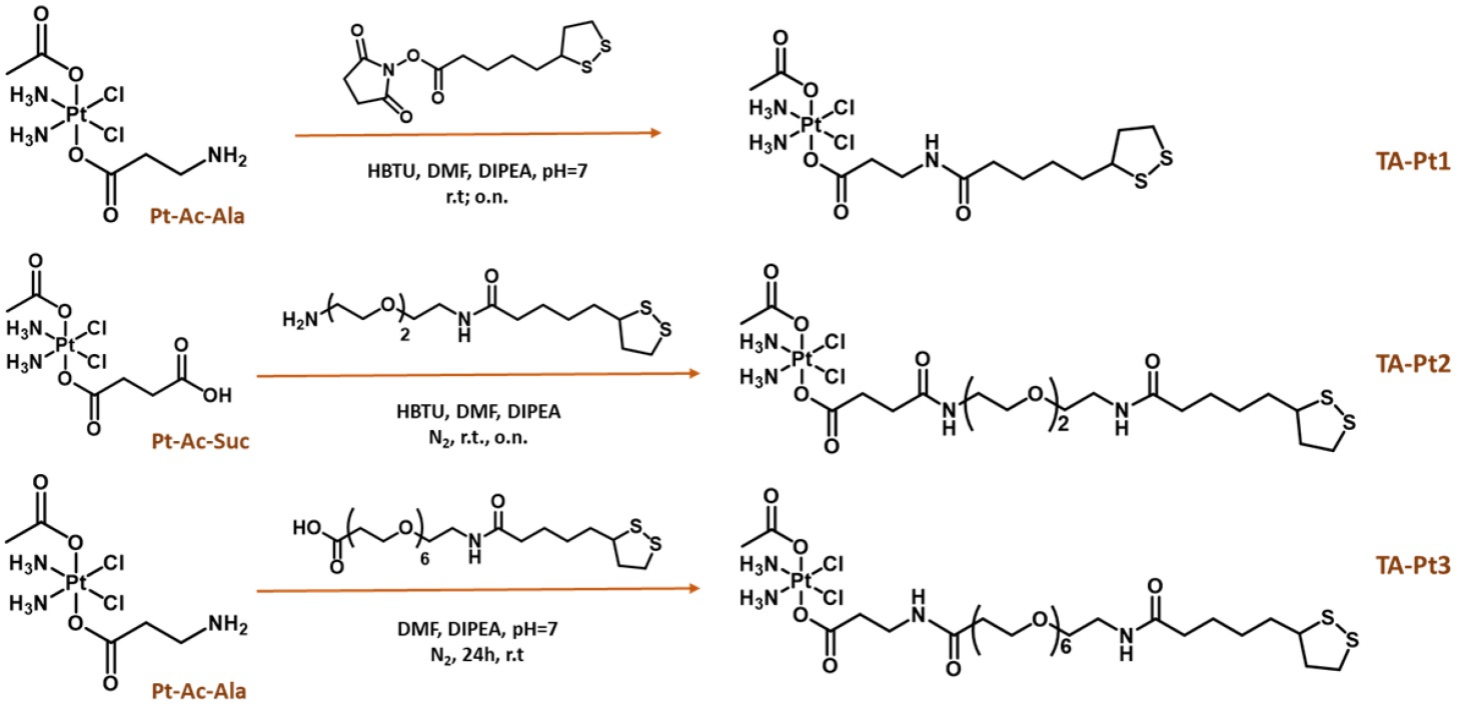
Schematic synthesis of thioctic acid-containing Pt(IV) prodrugs.

We have synthesized three thioctic acid derivatives containing the Pt(IV) prodrug (**TA-Pt, Pt = Pt1-Pt3**), carrying or not a PEGylated linker (PEG2 or PEG6) between the thioctic acid unit and the Pt(IV) core, as shown in Scheme 2. By introducing different linkers, we have anticipated that they might influence the colloidal stability and solubility of the **AuNP-BBN** nanoparticles following their functionalization with the Pt(IV) prodrugs, as well as the amount of loaded Pt(IV) prodrug. Ultimately, these features could affect the biological performance of the final Pt-containing AuNPs. To obtain these thioctic acid derivatives of the Pt(IV) prodrugs, we have started from two different Pt precursors, **Pt-Ac-Ala** and **Pt-Ac-Suc**. **Pt-Ac-Ala** was first used for a direct coupling with thioctic acid through an amidation reaction involving the terminal amino group from the axial alanine ligand and the *N*-hydroxysuccinimide activated ester of thioctic acid (**TA-NHS**), which led to the formation of the conjugate **TA-Pt1**. **Pt-Ac-Ala** has been also used to obtain **TA-Pt3**, containing a PEG6 linker between the Pt(IV) complex and the thioctic acid unit. The synthesis of **TA-Pt3** was achieved by reacting **Pt-Ac-Ala** with the appropriate PEGylated derivative of thioctic acid, upon activation with 2-(1H-benzotriazol-1-yl)-1,1,3,3-tetramethyluronium hexafluorophosphate (HBTU) in the presence of N,N-diisopropylethyl amine (DIPEA). **TA-Pt2** carrying a PEG2 linker was obtained similarly by reacting **Pt-Ac-Su** with a PEG2 derivative of thioctic acid. The synthesis of these pegylated thioctic acid derivatives has been detailed in the SI (Figure S1).

**Figure 2:**
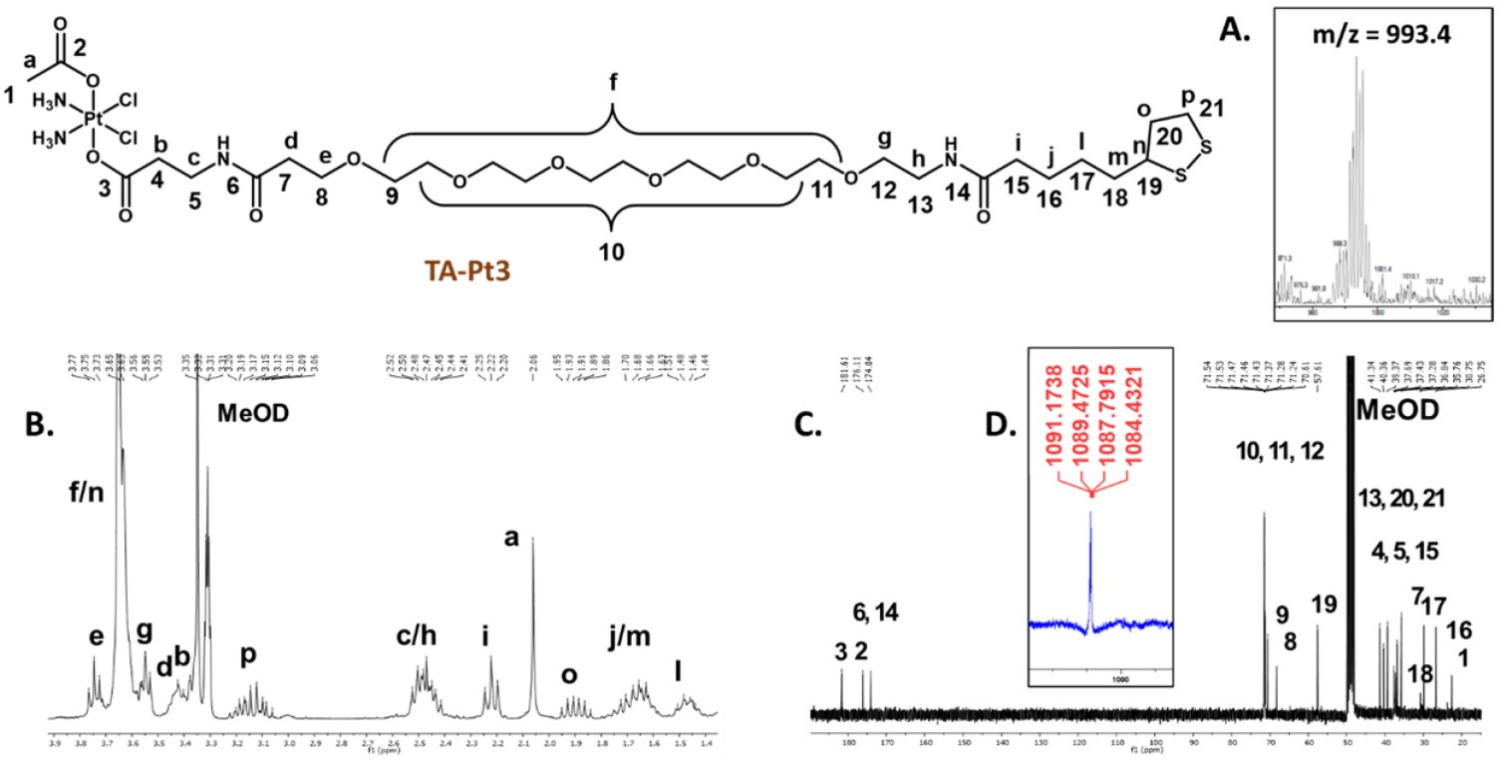
The thioctic acid-containing Pt(IV) prodrugs were characterized by ESI-MS (**A**) and multinuclear ^1^H, ^13^C and^ 195^Pt NMR analysis in MeOD (**B**, **C** and **D**), as exemplified for derivative **TA-Pt3**.

Compounds **TA-Pt** (**Pt**= **Pt1**-**Pt3**) were obtained in rather low isolated yields (6.1-14.3 %), which is essentially due to the difficulty of their separation from the corresponding unreacted precursors requiring a semi-preparative HPLC purification. After HPLC purification, all compounds were obtained in the form of yellowish oils that are soluble in polar organic solvents, such as methanol, DMF and DMSO. Their characterization was done by ESI-MS, multinuclear NMR (^1^H, ^13^C^,^ and ^195^Pt) and analytical HPLC, as exemplified in Figure 2. The ESI(+) mass spectra of **TA-Pt** confirmed the formation of the desired conjugates being observed prominent molecular ion [M]^+^ peaks and [M+Na]^+^ ions in the case of **TA-Pt1** and **TA-Pt2**, while for **TA-Pt3** only the [M+Na]^+^ ion was found in the spectra (Figure 2A and Figure S2). All of these peaks presented isotope distributions in agreement with the proposed formulations and consistent with the presence of Pt atoms.

The characterization of the compounds by multinuclear NMR (Figure 2B, C, and D) and Figures S3-4) also corroborated their molecular structures, with the assignment of the ^1^H and ^13^C resonances being supported by 2D homo- and heteronuclear NMR experiments (COSY and HSQC, respectively). In particular, each ^1^H NMR spectrum shows the characteristic multiplet of the α-proton from the 1,2-dithiolane ring 40, in the range 3.58-3.61 ppm, and a singlet from the methyl protons of the acetate ligand, in the range 1.90-2.06 ppm, thus confirming the presence of the thioctic acid part and Pt(IV) complex, respectively. The ^1^H and ^13^C NMR data are also consistent with the presence of the different linkers, namely in which concerns the ^13^C=O signals from the respective carboxylate and amide groups, appearing between 171.50 and 181.33 ppm. The ^195^Pt NMR chemical shifts of compounds **TA-Pt** (**Pt= Pt1-Pt3**), around 1100-1200 ppm, are consistent with the presence of Pt(IV) complexes with the “PtCl_2_N_2_O_2_” core 41, 42.

### Synthesis and characterization of BBN-containing AuNPs carrying Pt(IV) prodrugs

**Figure 3:**
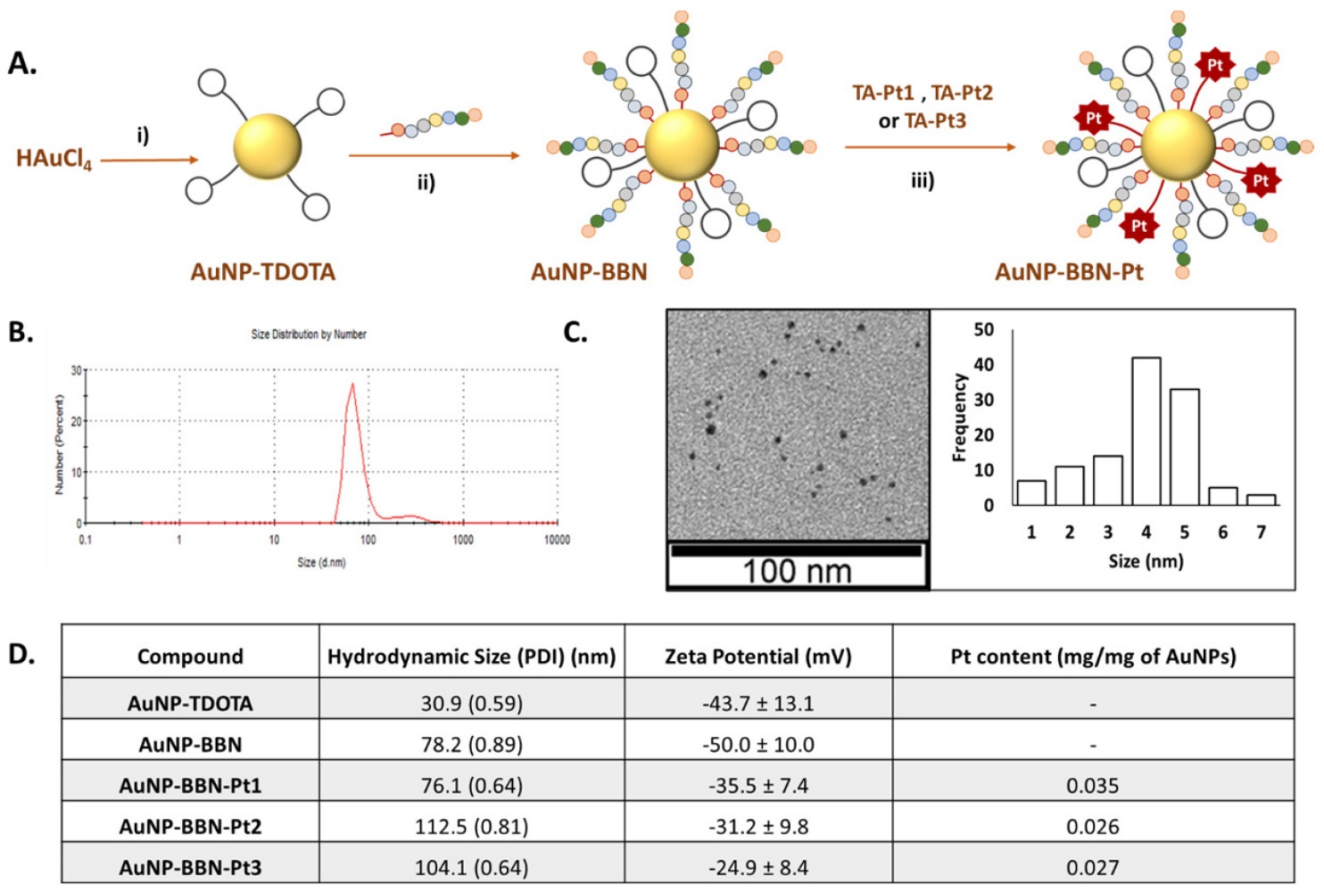
Schematic synthesis of the Pt(IV) prodrug-containing AuNPs: i) **TDOTA**, NaBH_4_, MeOH/H_2_O, 1 h at room temperature (r.t.).; ii) **TA-BBN**, MeOH/H_2_O, 2 h at r.t.; iii) **TA-Pt (Pt = Pt1, Pt2)** in H_2_O/DMSO or **TA-Pt3** in H_2_O/MeOH, overnight at r.t. (**A**). DLS analysis and TEM imaging with size histogram of **AuNP-BBN-Pt1** (**B** and **C**). Hydrodynamic size, zeta-potential and Pt content of the studied AuNPs are also reported (**D**).

The functionalization of **AuNP-BBN** with the different thioctic acid derivatives of the prodrug (**TA-Pt** (**Pt= Pt1-Pt3**)) was performed in a similar way to that we described earlier for the functionalization of **AuNP-TDOTA** with the BBN peptide 28. Thus, the Pt-containing nanoparticles,** AuNP-BBN-Pt** (**Pt** = **Pt1**-**Pt3**), were synthesized at room temperature by an overnight reaction of **AuNP-BBN** with the desired **TA-Pt** compound (1:2 molar ratio) in DMSO/water (**AuNP-BBN-Pt1** and **AuNP-BBN-Pt2**) or methanol/water (**AuNP-BBN-Pt1**) mixtures (Figure 3A). The **AuNP-BBN-Pt** were recovered by ultra-centrifugation, washed with water, and lyophilized. The characterization of these Pt-containing AuNPs involved the common techniques used to assess the physico-chemical properties of nanoparticles, such as UV-visible spectroscopy, TEM analysis and zeta potential measurements (Figure 3B-D, Figures S3-4), as well as the determination of their Pt content by ICP-MS. The most relevant physico-chemical data collected for **AuNP-BBN-Pt** are presented in the Table inserted in Figure 3D**,** in comparison to the parental **AuNP-BBN**.

The TEM analysis (Figure 3C and Figure S5) confirmed that the different nanoparticles have a small-sized core (4-5 nm), as we have previously described for **AuNP-BBN** 28-30. The DLS and zeta potential measurements showed that the functionalization of the AuNPs with the Pt(IV) prodrugs is accompanied by an increase of their hydrodynamic size with less negative zeta potential values, which is more significant for those carrying the PEGylated linker (hydrodynamic sizes in the range 104.1-112.5 nm; zeta potential values ranging from -31.2 to -24.9 mV) compared with those carrying uniquely the thioctic acid moiety (hydrodynamic size of 76.1 nm and zeta potential value of -35.5 mV). This trend certainly reflects the greatest molecular size of the PEGylated derivatives. The ICP-MS measurements showed that the Pt content of the final AuNPs varies in the range 0.026 to 0.035 mg of Pt per mg of nanoparticle. The Pt concentration measured for **AuNP-BBN-Pt1** (0.035 mg/mg AuNP) is higher than that determined for the PEGylated congeners (**AuNP-BBN-Pt2**, 0.026 mg/mg AuNP; **AuNP-BBN-Pt3**, 0.027 mg/mg AuNP), most probably due to the higher steric requirements of the PEGylated Pt(IV) precursors.

### Radiolabeling and *In vitro* Stability Studies

**Figure 4:**
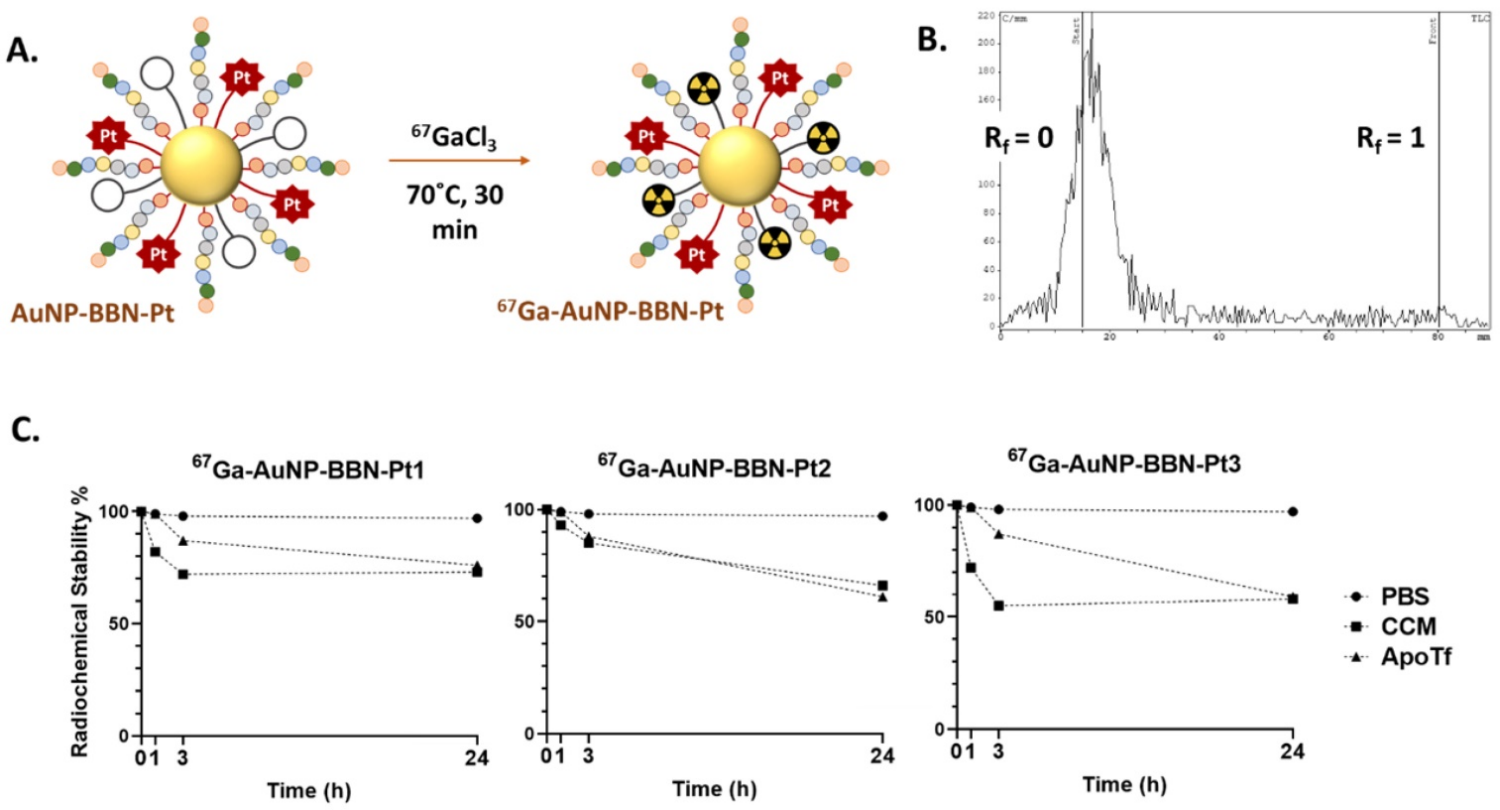
** A.** Schematic synthesis of the ^67^Ga-labeled AuNPs. **B.** iTLC radiochromatogram of **^67^Ga-AuNP-BBN-Pt1**. **C.** *In vitro* stability studies of the ^67^Ga-labeled AuNPs containing Pt(IV) prodrugs, in PBS 0.1 M, cell culture medium (CCM) and in the presence of apo-transferrin (ApoTf), at 37 °C.

The different Pt-containing AuNPs were labelled with ^67^Ga using the same approach that we have previously described for the radiolabeling of the congener NPs without the Pt(IV) prodrugs. Briefly, the desired nanoparticles were reacted with ^67^GaCl_3_ at 70 ºC for 30 min in ammonium acetate buffer at pH=7 (Figure 4A). After ultrafiltration, the radiolabeled AuNPs were recovered in high radiochemical yield and with high radiochemical purity (> 95%) as verified by radio-TLC analysis (Figure 4B and Figure S6).

The *in vitro* radiochemical stability of the different ^67^Ga-labeled AuNPs was evaluated under physiological conditions (0.1 M PBS) and in the presence of cell culture medium (CCM) and apo-transferrin (ApoTf). For this purpose, the AuNPs were incubated with each challenging media at 37 °C up to 24 h, and their radiochemical purity was assessed by radioTLC (Figure 4C and Figure S6). All radiolabeled nanoparticles presented excellent radiochemical stability under physiological conditions without release of coordinated ^67^Ga. However, their *in vitro* stability was somehow lower in the presence of cell medium or *apo*-transferrin, in particular for the NPs functionalized with the PEGylated derivatives. Owing to their physicochemical similarities with Fe^3+^, Ga^3+^ ions can readily bind to transferrin, which is an iron transport protein with a relatively high concentration (ca. 2.5 mg/mL) in human blood 43. Even so, **^67^Ga-AuNP-BBN-Pt1** displayed a good stability (radiochemical purity *ca.* 75 %) after 24 h of incubation with cell medium or *apo*-transferrin, showing a substantial resistance to ^67^Ga^3+^ trans-chelation processes involving the components of the cell medium or *apo*-transferrin. For the NPs functionalized with PEGylated derivatives, **^67^Ga-AuNP-BBN-Pt2** and **^67^Ga-AuNP-BBN-Pt3**, a more significant release of ^67^Ga was observed but still 60% of the ^67^Ga activity remained associated with the NPs after 24 h of incubation with these two challenging media. Overall, these favorable results encouraged us to use the ^67^Ga-labeled AuNPs for a prompt assessment of their cellular uptake in GPPR+ cell lines and their tumor uptake and retention in corresponding tumor xenografts based on quantitative radiometric measurements and microSPECT imaging studies, as described below.

### Cellular Studies of the BBN-containing AuNPs carrying Pt(IV) prodrugs

Initial *in vitro* studies were performed to enlighten how the different linkers used to attach the Pt(IV) prodrugs to the AuNPs surface would influence their anticancer properties and comprised the assessment of their binding affinity, the determination of the cellular uptake and internalization as well as the evaluation of their cytotoxicity using the GRPR-positive human tumor cell line PC3. The aim of such preliminary evaluation was to select the most promising **AuNP-BBN-Pt** nanoparticle for further and more detailed cellular and animal studies.

#### Binding Affinity

The binding affinity of the **AuNP-BBN-Pt** towards the GRPR was determined by a competitive binding assay using the GRPR-specific radioligand [^125^I-Tyr4]BBN and the GRPR+ cell line PC3. The curves obtained allowed the determination of the IC_50_ values of the **AuNP-BBN-Pt** nanoparticles (Figure S7). The compound **AuNP-BBN-Pt1** has the highest binding affinity, with an IC_50_ value of 0.055 μg mL^-1^ of AuNPs, followed by **AuNP-BBN-Pt3** and** AuNP-BBN-Pt2** with IC_50_ values of 0.096 and 0.160 μg mL^-1^, respectively (see the Table inserted in Figure 6B). The IC_50_ value measured for **AuNP-BBN-Pt1** is similar to that we have reported for the parental **AuNP-BBN** (IC_50_ = 0.045 μg mL^-1^) 28, showing that the functionalization of the AuNPs with the Pt(IV) prodrug did not interfere much with their interaction with the GRPR.

#### Cellular uptake and Cytotoxicity in a GRPR-positive Cell line

**Figure 5:**
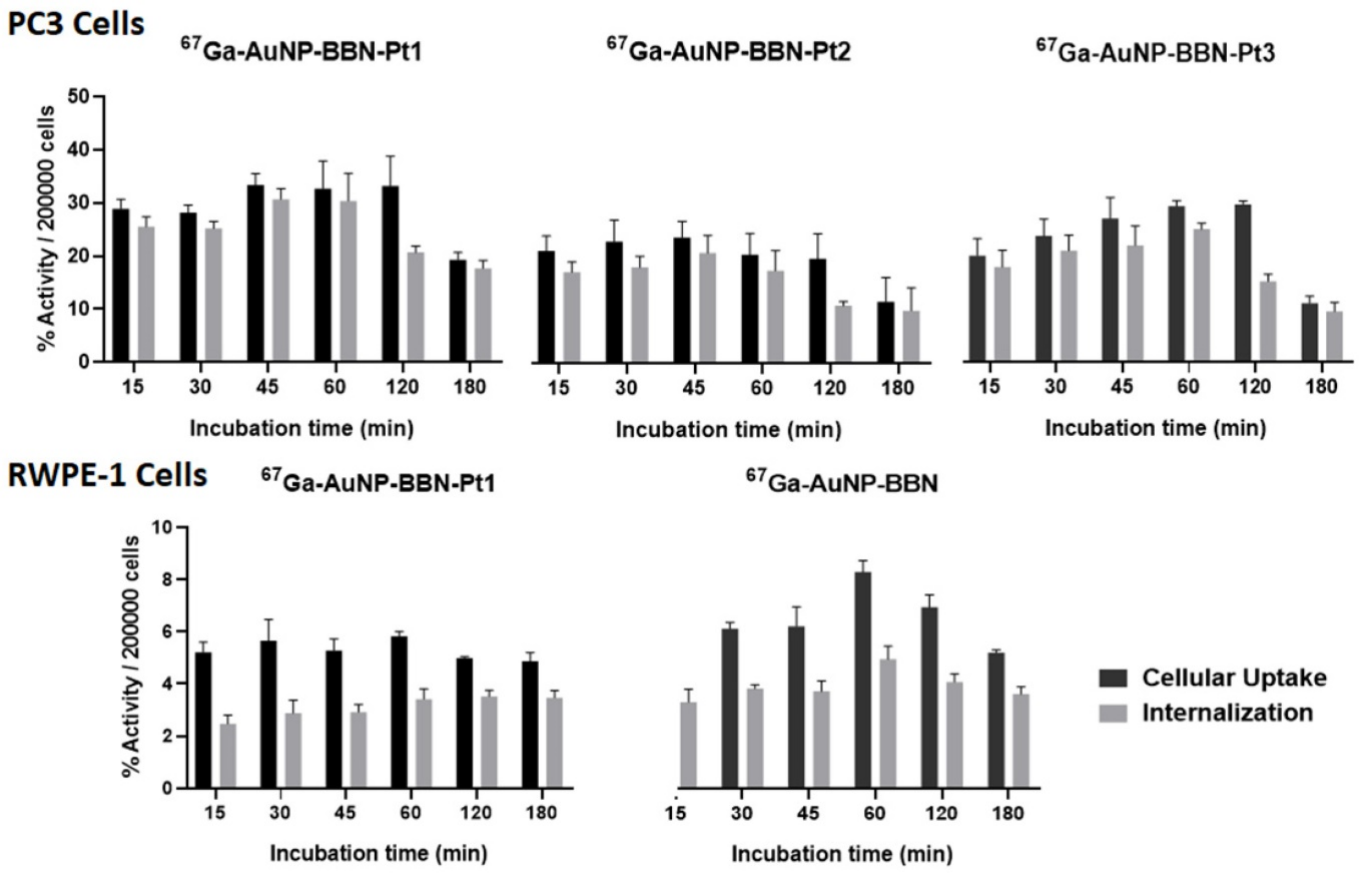
Cellular uptake and internalization of the radiolabeled nanoparticles in the GRPR+ human prostate cancer PC3 cell line and in the normal RWPE-1 prostate cell line with a low level of GRPR expression.

The ^67^Ga-labeled congeners **^67^Ga**-**AuNP-BBN-Pt** (**Pt** = **Pt1**-**Pt3**) were used for the cellular uptake and internalization studies. As seen from the results showed in Figure 5, all the ^67^Ga-labeled AuNPs presented a fast kinetics of cellular uptake with an extensive internalization upon incubation at 37 ºC with the tumor cells.

For all the AuNPs, the percentage of the activity associated to the cells and corresponding to internalized activity spanned between 31 and 10% for the tested time points from 15 min to 3 h. The internalization rate reached its maximum value after 1 h of incubation with each AuNPs and, thereafter, suffered a slight to moderate decrease. **AuNP-BBN-Pt1** without any PEGylated linker showed greater cellular uptake and internalization when compared to the PEGylated congeners **AuNP-BBN-Pt2** and** AuNP-BBN-Pt3**, which eventually reflects its highest binding affinity towards the GRPR.

The cytotoxic activity of **AuNP-BBN-Pt** (**Pt** = **Pt1**-**Pt3**) was evaluated in the PC3 cell line by incubating the cells with increasing concentrations of the different AuNPs for 72 h at 37 °C and assessment of the cellular viability by the 1-(4,5-dimethylthiazol-2-yl)-2,5-diphenyl tetrazolium] (MTT) assay. The assays were performed under the same conditions for cisplatin and for the parental **AuNP-TDOTA** and **AuNP-BBN** to investigate possible contributions of the NPs core and BBN peptide for the cytotoxicity of the Pt-containing nanoparticles. For this study, the Au concentrations studied for the parental NPs were equivalent to those involved in the studies with the Pt-containing **AuNP-BBN-Pt**.

**Figure 6:**
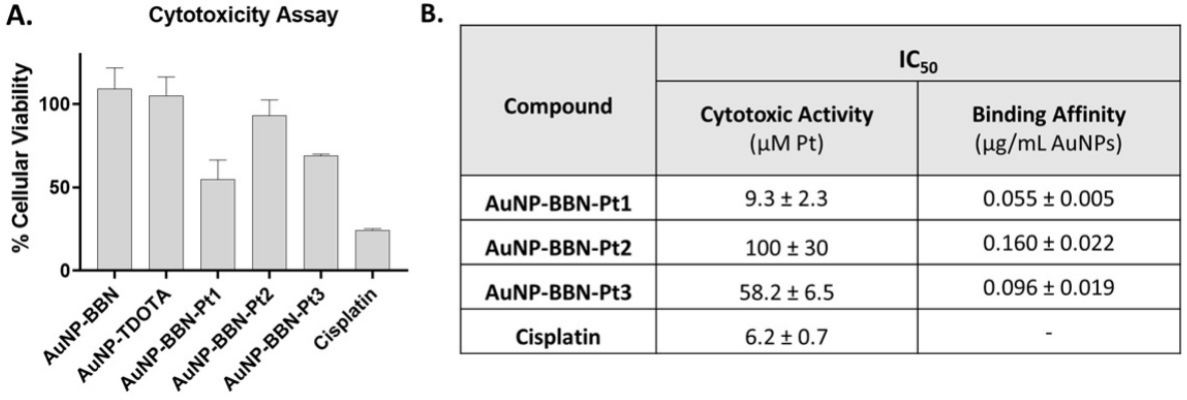
**A.** Comparison of the viability of PC3 cells exposed to **AuNP-BBN-Pt** (**Pt = Pt1-Pt3**) and cisplatin at 20 µM Pt concentration (or in the corresponding Au concentration for **AuNP-BBN** and **AuNP-TDOTA**) in PC3 cells, after incubation at 37 ºC for 72 h. **B.** The IC_50_ values (Cytotoxic Activity and Binding Affinity) determined in PC3 cells for the Pt(IV) prodrug-containing AuNPs and cisplatin.

As exemplified in Figure 6A, **AuNP-TDOTA** and **AuNP-BBN** have a negligible or weak effect on the cell viability of PC3 cells when compared with the congener **AuNP-BBN-Pt1** applied at the same gold concentration. Since the cell viability relative to the control (i.e., cells exposed uniquely to the culture medium) of the tumor cells exposed to **AuNP-TDOTA** and **AuNP-BBN** was well above 50% in the entire range of tested concentrations, the respective IC_50_ values (i.e., concentration that reduces the growth by 50%) were not determined. On the other hand, **AuNP-BBN-Pt** (**Pt** = **Pt1**-**Pt3**) exhibited a stronger cytotoxicity that allowed the determination of the IC_50_ values (expressed as Pt concentration) in the PC3 cell line, based on the measured inhibition of growth (%). The results are presented in the Table inserted in Figure 6B in comparison with cisplatin.

The presence of the PEGylated linkers had a marked influence on the cytotoxic activity of the **AuNP-BBN-Pt** nanoparticles, with **AuNP-BBN-Pt1** (IC_50_ = 9.3±2.3 μM of Pt) being considerably more cytotoxic than the PEGylated congeners **AuNP-BBN-Pt2** (IC_50_ =100±30 μM of Pt) and **AuNP-BBB-Pt2** (IC_50_ = 58.2±6.5 μM of Pt). The IC_50_ value measured for **AuNP-BBN-Pt1** in PC3 cells is relatively similar to that exhibited by cisplatin (IC_50_ = 6.2±2.3 μM).

In some way, the trend of the cytotoxic activity of **AuNP-BBN-Pt** (**Pt = Pt1-Pt3**) in the PC3 cells is consistent with the cellular uptake and internalization data obtained using the ^67^Ga-labeled congeners, as discussed above. **AuNP-BBN-Pt1** showed a higher internalization in the PC3 cells when compared to the PEGylated congeners **AuNP-BBN-Pt2** and **AuNP-BBN-Pt3**, which can justify the highest cytotoxicity of the former.

**Figure 7:**
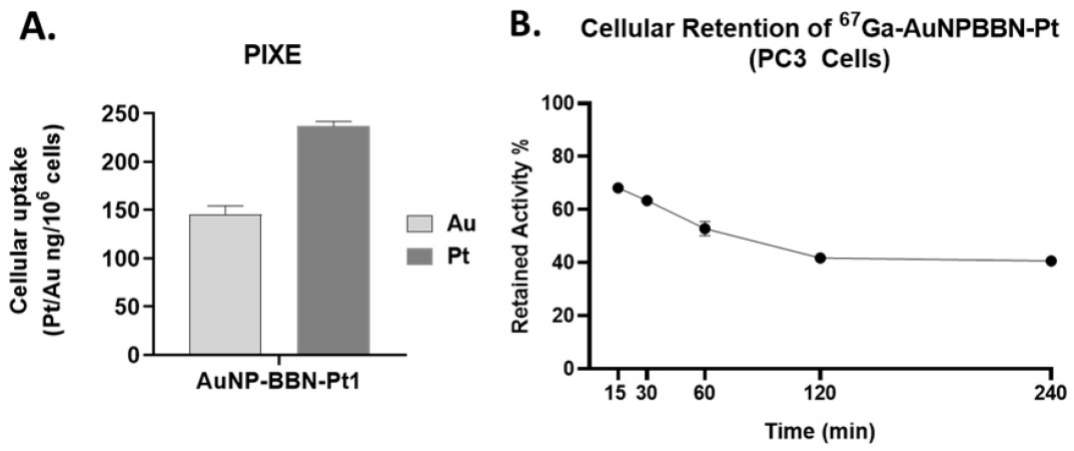
**A.** Quantification of Au and Pt inside the cells after incubation of PC3 cells with **AuNP-BBN-Pt1** at 37 ºC for 24 h.** B.** Cellular retention of the derivative **^67^Ga-AuNP-BBN-Pt1**.

Altogether, the above described cellular studies led us to identify **AuNP-BBN-Pt1** as the most promising AuNP of the series for the specific delivery of Pt(IV) prodrugs to GRPR+ tumors. Thus, we have further characterized its cellular uptake through the quantification of Au and Pt by particle-induced X-ray emission (PIXE) analysis, after incubation of the cells with **AuNP-BBN-Pt1** at 37 ºC for 24 h. Interestingly, the results have shown that there is a greater accumulation of Pt than Au in the tumor cells, being the Pt uptake roughly twice that of Au uptake (Figure 7A). The greatest Pt accumulation eventually reflects the release of cisplatin from the nanoplatforms, upon intracellular reduction of Pt(IV) to Pt(II). Then, the released cisplatin is trapped inside the cells through interaction with cytoplasmic proteins and/or nuclear DNA. On its turn, the gold nanoparticles themselves can be effluxed from the cells, as we have previously observed for **^67^Ga-AuNP-BBN** in PC3 cells 28. As shown in Figure 7B, the congener **^67^Ga-AuNP-BBN-Pt1** also undergoes such washout processes at a similar efflux rate. Thus, the combination of cisplatin release inside the cells with the AuNPs efflux from the cells can certainly explain the greatest cellular accumulation found for Pt compared to Au in the PC3 tumor cells.

#### Cytotoxicity Selectivity and Mechanisms of Cell Death: AuNP-BBN-Pt1 versus Cisplatin

As mentioned above, the cytotoxicity and cellular uptake studies of the different Pt-containing AuNPs prompted us to consider **AuNP-BBN-Pt1** as the most promising AuNP for the specific delivery of Pt(IV) prodrugs to GRPR+ tumors, thus deserving further evaluation in cellular and animal models in comparison with cisplatin.

In the tested GRPR+ tumor cell line, **AuNP-BBN-Pt1** presented micromolar IC_50_ values relatively similar to those exhibited by cisplatin. These results differ from those reported by some of us for non-targeted silica nanoparticles carrying the same type of Pt(IV) prodrugs, which were much more active than cisplatin in A2780 ovarian cancer cells 31. However, the results obtained for **AuNP-BBN-Pt1** are somewhat in tune with those reported by other authors for targeted AuNPs used to deliver related Pt(IV) prodrugs, for which a cytotoxicity relatively similar to that of cisplatin was observed, namely in PC3 cells 18, 23.

**Figure 8:**
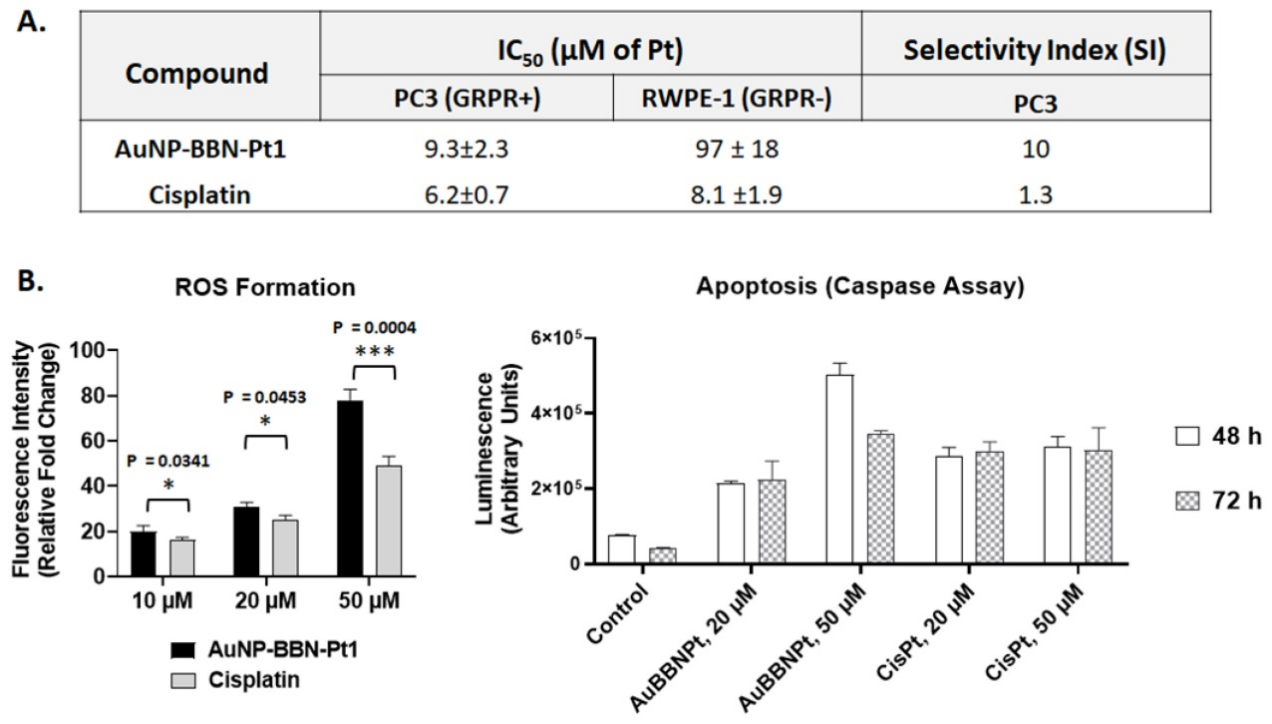
Comparison between the compound **AuNP-BBN-Pt1** and cisplatin in terms of **A.** selectivity toward GRPR+ cell lines and **B.** mechanisms of cytotoxicity determined through ROS formation and apoptosis assay (both for **AuNP-BBN-Pt1** and cisplatin the values are expressed as Pt concentration).

In fact, our major goal was to improve the selectivity of the cytotoxic action of the designed Pt-containing AuNPs and not necessarily to augment their cytotoxicity, which could be a source of undesired side effects often caused by cisplatin. To address this issue, we have compared the cytotoxic activity of **AuNP-BBN-Pt1** and cisplatin in the normal prostate epithelial cell line (RWPE-1) and in the tumor cell line. Most importantly, the RWPE-1 cell line is known to express GRPR in a much lower level than the tumor congener 44, and therefore suitable to validate the designed GRPR-targeted delivery of Pt(IV) prodrugs. The IC_50_ values determined for **AuNP-BBN-Pt1** and cisplatin in PC3 and RWPE-1 cells are compared in the table inserted in Figure 8A, together with the selectivity index (SI) calculated for both compounds in each tumor cell line. The selectivity index was calculated from the ratio of the IC_50_ values of the compounds in tumor cell lines and in the RWPE-1 cell line. The selectivity index is an indicator of the selectivity of a given compound between normal and cancer cells, corresponding a higher selectivity index to a greater selectivity 45.

The **AuNP-BBN-Pt1** is much more active in the tumor cell line than in the non-cancerous RWPE-1, presenting a high selective index of 10. This value is considerably higher than the SI value of 1.3 exhibited by cisplatin for the same cell lines. The highest selectivity of **AuNP-BBN-Pt1** is certainly due to the presence of the targeting BBN peptide that promotes a preferential uptake in the GRPR-positive cell line compared to the RWPE-1 cell line, as we have previously confirmed for the parental **AuNP-BBN** by PIXE analysis of the Au content in the cells 30. Cellular uptake and internalization studies in RWPE-1 cells confirmed that **^67^Ga-AuNP-BBN-Pt1** is less prone to internalization in this cell line compared to the PC3 cell line (Figure 5).

Lastly, to have some insight into the mechanisms involved in the cytotoxic activity of **AuNP-BBN-Pt1**, we have studied how exposure of the PC3 tumoral cells to these AuNPs influences the production of reactive oxygen species (ROS) and the induction of apoptosis. The studies were carried out in parallel with cisplatin, under the same conditions and for the same range of Pt concentrations, as cisplatin is generally considered to increase the generation of intracellular ROS that can damage different cellular structures, such as DNA, proteins, and lipids, leading to apoptosis 46. The results of the ROS and apoptosis assays are presented in Figures 8B and 8C, respectively.

As can be seen in Figure 8B, **AuNP-BBN-Pt1** and cisplatin enhanced ROS production at similar extent in the range of concentrations tested (10-50 μM of Pt). The only exception was for the highest Pt concentration (50 μM), with **AuNP-BBN-Pt1** causing a greater ROS production than cisplatin. We explored the use of the active form of caspase-3 and 7 for the detection of apoptotic events induced by **AuNP-BBN-Pt1** and cisplatin. Caspases are a family of cysteine proteases that regulate ordered processes such as apoptosis. Among them, caspase-3 and caspase-7 are considered executioners of the apoptotic pathways and can be used in cellular assays to identify modulators of the cell death cascade 47. As shown in Figure 8B, the ability of **AuNP-BBN-Pt1** to activate caspase-3/7 *in vitro* is superior to cisplatin. Apparently, it is not directly correlated with its ability to induce cell death when compared to the reference drug, according to the determined IC_50_ values. All in all, these results pinpoint that the biological effects involved in the cell death caused by **AuNP-BBN-Pt1** in the GRPR-positive PC3 cell line are similar to those induced by cisplatin. This seems to be consistent with the release of the active drug cisplatin from the nanoplatform, following the reduction of the Pt(IV) prodrug in the more reducing intracellular milieu of the tumor cells.

### Biodistribution and Small Animal Imaging Studies

We have thought that the use of the designed GRPR-targeted AuNPs carrying activatable Pt(IV) prodrugs within an intratumoral administration approach would further enhance the selectivity of the devised anticancer strategy, avoiding the undesired side effects inherent to cancer treatment with cisplatin. Furthermore, such a strategy allows a molecularly targeted image-guided approach to follow the local delivery of the cytotoxic drugs, being particularly useful for aggressive GRPR-positive solid tumors. In a way, this approach is related to a kind of nano-brachytherapy, using nanoseeds functionalized with tumor-specific biomolecules and injected intratumorally, which in recent year started to be envisaged for the delivery of therapeutic radionuclides as an alternative to classical brachytherapy seeds 48. Most importantly, these injectable nanoseeds can reduce the trauma inherent to surgical implantation of the brachytherapy seeds in clinical use, namely in the case of prostate cancer.

Thus, we have studied the biodistribution of **^67^Ga-AuNP-BBN-Pt1** in a PC3 xenograft model, upon bolus intratumoral administration of radiolabeled AuNPs. The study was carried out 1, 24 and 72 h post-administration and comprised the measurement of the ^67^Ga activity retained in different organs and tissues, as well as in the tumor, after euthanizing the mice and appropriate excision of the different tissues (Table S1). To assess the Pt and Au amount retained in tumors overtime, the amount of platinum and gold was determined by ICP-MS analysis after complete decay of the ^67^Ga activity. For comparison, xenograft-bearing mice were intratumorally injected with a cisplatin solution with approximately the same Pt concentration and sacrificed at the same time points.

**Figure 9:**
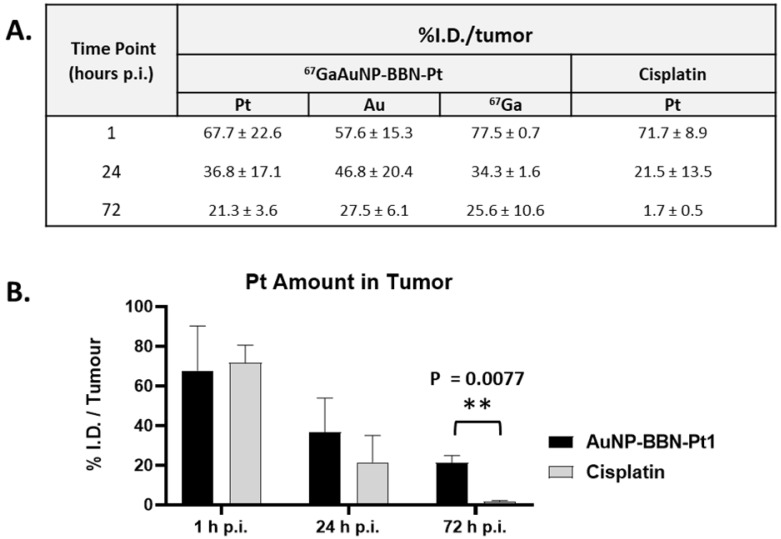
**A.** Tumor uptake (% I.D.) of platinum, gold and radioactive gallium at 1, 24 and 72 h after intratumoral injection.** B.** Comparison of the overtime intratumoral retention of Pt between **AuNP-BBN-Pt1** and cisplatin.

The results were expressed as % I.D. and are presented in the table and graph inserted in Figure 9. Data show that for **AuNP-BBN-Pt1** more than 20% of the injected dose of platinum and gold remain in the tumor up to 72 h after administration, which is in clear contrast to the result obtained for the intratumoral administration of cisplatin. In this case, a significant washout of Pt occurred after 72 h of administration with a tumor retention inferior to 2% of the injected dose. This finding is a favorable feature for the *in vivo* anticancer action of **AuNP-BBN-Pt1** due to the more prolonged retention allowing a steady release of the active cisplatin drug.

**Figure 10:**
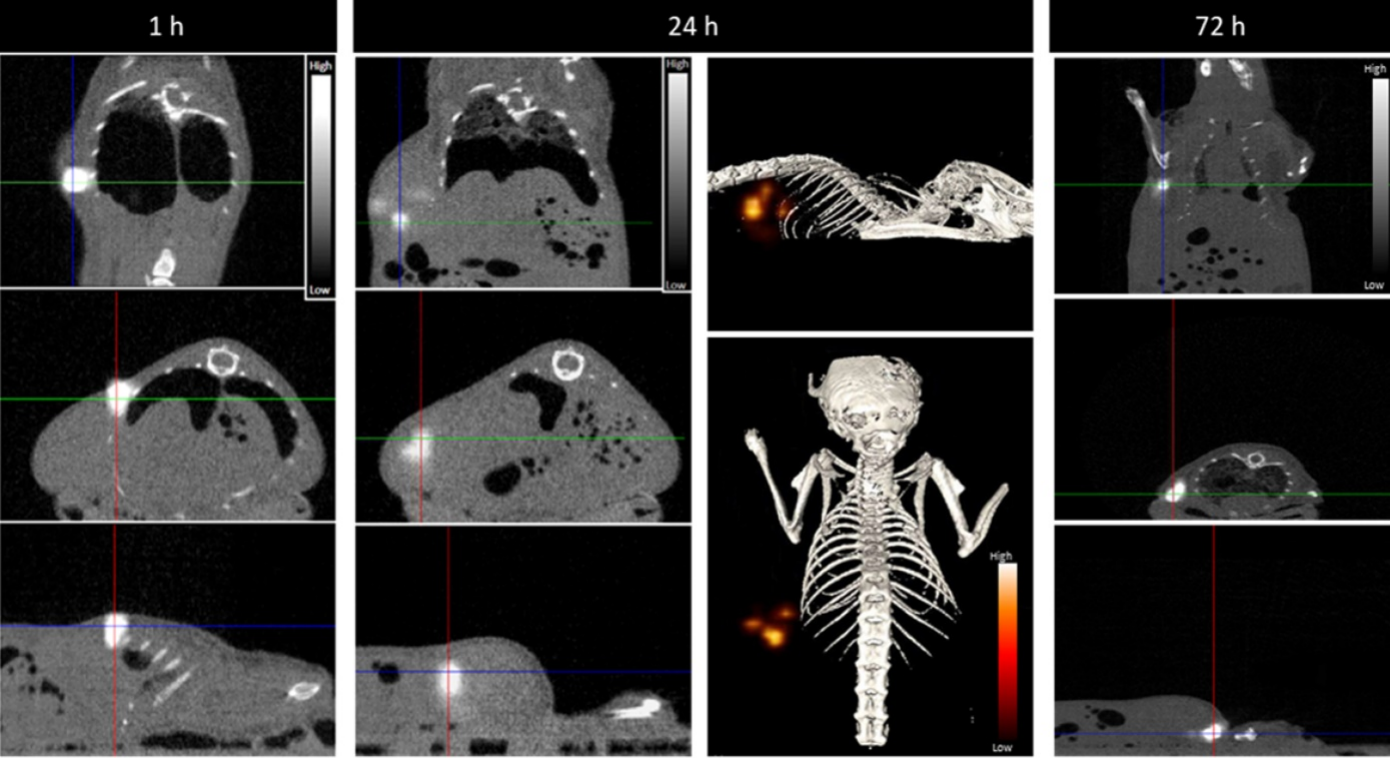
Serial SPECT/CT imaging at 1, 24, and 72 h after intratumoral injection of **^67^Ga-AuNP-BBN-Pt1**. The gray scale images represent the registered SPECT-CT slices at indicated times, respectively, the coronal (top), transaxial (center) and sagittal (bottom) planes. The color scale images represent a 3D view of the ^67^Ga activity, reflecting the tissue distribution studies. Intensity bars represent a range of intensities (arbitrary units) for all panels, which have been normalized to same intensity scale.

Most relevantly, the percentages of ^67^Ga, Au, and Pt retained in the tumor, relative to the injected dose of each radioactive or natural metal, are rather comparable between them for each post-injection time (1, 24 and 72 h). These results indicate the feasibility of the intended image-guided drug delivery approach, since the radioactive tag is retained similarly in the tumors as the AuNPs and carried Pt. To further validate this possibility, we performed microSPECT imaging studies in mice with PC3 tumor that were intratumorally injected with **^67^Ga-AuNP-BBN-Pt1.** Then, microSPECT-CT images were acquired after 1, 24 and 72 h post-injection, as detailed in the experimental section. For the different time points, the transaxial, coronal, and sagittal views obtained around the tumor are presented in Figure 10, together with a 3D image for the 24 h after injection time. As in the case of *ex vivo* biodistribution studies, the microSPECT-CT study showed a long retention of **^67^Ga-AuNP-BBN-Pt1** at the tumor site and negligible distribution in surrounding organs and tissues, even after 72 h of injection.

## Conclusions

The work reported herein demonstrates the ability of GRPR-targeted AuNPs for the specific delivery of Pt(IV) prodrugs to prostate cancer PC3 cells, within an image-guided and theranostic approach. In particular, **AuNP-BBN-Pt1** stood out as the most promising nanoparticles, displaying the highest cytotoxic activity with a favorable selective index for PC3 cells. These results are most likely attributed to their higher GRPR binding affinity and cellular internalization compared with the PEGylated AuNP congeners. Furthermore, **^67^Ga-AuNP-BBN-Pt1** shows optimal prolonged retention in tumor at 72 h p.i., as verified by biodistribution and microSPECT imaging studies, together with suitable *in vivo* stability. In general, these results call for further investigation of these **AuNP-BBN-Pt1** nanoparticles to thoroughly assess their potential for image-guided delivery of Pt(IV) prodrugs to GRPR+ tumors. For such studies, the designed multifunctional AuNPs offer the possibility to replace the imaging radionuclide with therapeutic ones (e.g., ^177^Lu or ^225^Ac), thus allowing the simultaneous delivery of cytotoxic drugs and therapeutic medical radionuclides that might help to circumvent chemoresistance and radioresistance issues. Thus, we are planning therapeutic assays with **AuNP-BBN-Pt1** in PC3 xenografts that will be also extended to the ^177^Lu-labeled counterparts.

## Materials and Methods

### General Procedures

All chemicals and solvents were of reagent grade and were used without further purification, unless stated otherwise, and were commercially acquired from Aldrich Chemical Co. Solvents for high-performance liquid chromatography (HPLC) were HPLC-grade. For the preparation of aqueous solutions and for rinsing of gold nanoparticles, Milli-Q (DI) water (ρ < 18MΩ) was used. The **AuNP-BBN** nanoparticles were synthesized according to previously published methods 28. The synthesis of (*OC*‑6‑44)‑acetato(β-alaninato)diamminedichloridoplatinum(IV) (**Pt-Ala**), (*OC*‑6‑44)‑acetatodiamminedichlorido(3-((((9H-fluoren-9-yl)methoxy)carbonyl)amino)propanoato)platinum(IV) and (*OC*‑6‑44)‑acetatodiammine(3-carboxypropanoato)dichloridoplatinum(IV) (**Pt-COOH**) was performed as described in the literature 41. The HPLC purifications were performed in a Perkin-Elmer *series 200* equipment, using a Supelco Analytical Discovery Bio C18 column and the following HPLC method: Solvent A = H_2_O, Solvent B = CH_3_OH; 100% A (0 - 5 min), 100% B (5 - 25 min), 100% A (25 - 26 min), 100% A (26 - 30 min). ^67^GaCl_3_ was prepared from ^67^Ga-citrate (acquired from Mallinckrodt) following a protocol previously described 49.

### Chemistry

#### Synthesis and Characterization of Lipoic Derivatives of Pt(IV) Prodrugs

##### Synthesis of TA-Pt1

**Pt-Ala** (11.6 mg, 0.026 mmol) was dissolved in dried DMF. To the resulting solution, 7.3 mg (0.024 mmol) of the N-hydroxysuccinimide activated ester of thioctic acid (TA-NHS) and 10 µL (0.057 mmol) of DIPEA were added (final pH = 7). The mixture was stirred at room temperature overnight. Then, the compound was purified by HPLC. The collected fractions were freeze-dried, after removal of the organic solvent under vacuum, to afford **TA-Pt1** (1.9 mg, 0.003 mmol) as a yellow oil (Ƞ = 11.5%).

R_T_ (HPLC) **:** 22.15 min. ESI-MS (CH_3_OH): m/z: [C_13_H_27_Cl_2_N_3_O_5_PtS_2_H]^+^: calculated = 636.0; found = 636.2; [C_13_H_27_Cl_2_N_3_O_5_PtS_2_Na]^+^: calculated = 658.0; found = 658.2. ^1^H NMR[DMSO-d_6_, δ (ppm)]: 3.61 (m, 1H, CH); 3.21-3.10 (br, 4H, 2CH_2_); 2.42 (m, 2H, CH_2_); 2.33 (t, 2H, CH_2_); 2.06 (t, 2H, CH_2_); 1.91 (s, 3H, CH_3_); 1.86 (m, 2H, CH_2_); 1.65 (m, 2H, CH_2_); 1.56-1.49 (br, 4H, 2CH_2_); 1.35 (m, 2H, CH_2_). ^13^C NMR [DMSO-d_6_, δ (ppm)]: 178.75 (C=O); 178.53 (C=O); 172.24 (C=O); 56.34 (CH); 38.28 (CH_2_); 36.28 (CH_2_); 35.81 (CH_2_); 35.50 (CH_2_); 34.35 (CH_2_); 28.58 (CH_2_); 25.13 (CH_2_); 23.05 (CH_3_). ^195^Pt NMR [DMSO-d_6_, δ (ppm)]: 1200.

##### Synthesis of TA-PEG_2_-NH-BOC

50 mg (0.2 mmol) of **NH_2_-PEG_2_-NH-BOC** were dissolved in 4 mL of dried DMF; then, 45 µL (0.26 mmol) of DIPEA were added, followed by 64 mg (0.21 mmol) of TA-NHS. The mixture was stirred overnight at room temperature under N_2_. The solvent was evaporated under low pressure and the compound purified by HPLC. The collected fractions were freeze-dried, after removal of the organic solvent under vacuum, to afford **TA-PEG_2_-NH-BOC** (49.8 mg, 0.16 mmol) (Ƞ = 79%).

ESI-MS (CH_3_CN): m/z: [C_19_H_36_N_2_O_5_S_2_H]^+^: calculated = 437.2; found = 437.8. ^1^H NMR [CDCl_3_, δ (ppm)]: 3.49-3.56 (m, 8H, 4CH_2_ & 1H, CH_2_); 3.41 (t, 2H, CH_2_); 3.26 (br, 2H, CH_2_); 3.01-3.17 (m, 2H, CH_2_); 2.38 (m, 1H, CH); 2.17 (t, 2H, CH_2_); 1.85 (m, 1H, CH_2_); 1.57-1.68 (m, 4H, 2CH_2_); 1.40 (m, 9H, 3CH_3_ & 1H, CH_2_). ^13^C NMR [CDCl_3_, δ (ppm)]: 172.95 (C=O); 156.12 (C=O); 79.34 (C(CH_3_)); 70.42 (2CH_2_); 69.54 (2CH_2_); 56.31 (CH); 40.36 (CH_2_); 39.18 (CH_2_); 38.21 (CH_2_); 35.78 (CH_2_); 34.52 (CH_2_); 28.75 (3CH_3_); 28.39 (CH_2_); 25.95 (CH_2_).

##### Synthesis of TA-PEG_2_-NH_2_

68.7 mg (0.16 mmol) of **TA-PEG2-NH-BOC** were dissolved in a 2 mL solution of TFA/dichloromethane (1:1). The mixture was stirred at room temperature for 2 h and then the solvents were evaporated under low pressure. The crude was dissolved in methanol, and, after filtration, the solvent was removed under vacuum to afford **TA-PEG_2_-NH**_2_ (68.7 mg, 0.15 mmol) (Ƞ = 94%).

ESI-MS (CH_3_CN): m/z: [C_14_H_28_N_2_O_3_S_2_H]^+^: calculated = 337.2; found = 338.0. ^1^H NMR [CD_3_OD, δ (ppm)]: 3.61 - 3,74 (m, 4H, 2CH_2_ & 1H, CH); 3.56 (t, 2H, CH_2_); 3.38 (t, 2H, CH_2_); 3.05-3.23 (m, 4H, 2CH_2_); 2.47 (m, 1H, CH); 2.22 (t, 2H, CH_2_); 1.88 (m, 1H, CH_2_); 1.58-1.79 (m, 4H, 2CH_2_ & 1H, CH_2_); 1.47 (m, 2H, CH_2_).^ 13^C NMR [CD3OD, δ (ppm)]: 176.49 (C=O); 71.53 (2CH_2_); 70.72 (CH_2_); 68.20 (CH_2_); 57.66 (CH_2_); 41.47 (CH_2_); 40.67 (CH_2_); 40.10 (CH_2_); 39.79 (CH_2_); 37.00 (CH_2_); 36.19 (CH_2_); 30.06 (CH_2_); 27.27 (CH_2_); 26.72 (CH_2_).

##### Synthesis of TA-Pt2

**TA-PEG_2_-NH_2_**_ (_20 mg, 0.06 mmol) was dissolved in 3 mL of dried DMF. To the resulting solution, 22.76 mg (0.06 mmol) of HBTU were added, followed by 10.5 µL of DIPEA. Then, 28.5 mg (0.06 mmol) of **Pt-COOH** were added and the mixture was stirred overnight at room temperature under N_2_. Then, the compound was purified by HPLC. The collected fractions were freeze-dried, after removal of the organic solvent under vacuum, to afford **TA-Pt2** (2.9 mg, 0.0037 mmol) as a yellow oil (Ƞ = 6.1%).

R_T_ (HPLC): 22.12 min. ESI-MS (CH_3_OH/DMF): m/z: [C_20_H_40_Cl_2_N_4_O_8_PtS_2_H]^+^: calculated = 795.1; found = 795.3; [C_20_H_40_Cl_2_N_4_O_8_PtS_2_Na]^+^: calculated = 817.1; found = 817.2. ^1^H NMR [DMSO-d_6_, δ (ppm)]: 3.58 (m, 1H, CH); 3.39 (t, 2H, CH_2_); 3.32 (m, 4H, 2CH_2_); 3.20-3.09 (br, 8H, 4CH_2_); 2.44-2.36 (br, 3H, 2CH_2_); 2.28 (t, 2H, CH_2_); 2.06 (t, 2H, CH_2_); 1.90 (s, 3H, CH_3_); 1.85 (m, 2H, CH_2_); 1.67-1.47 (br, 6H; 3CH_2_); 1.33 (m, 2H, CH_2_). ^13^C NMR [DMSO-d_6_, δ (ppm)]: 180.01 (C=O); 178.18 (C=O); 172.10 (C=O); 171.52 (C=O); 69.52 (2CH_2_); 69.17 (CH_2_); 69.07 (CH_2_); 56.15 (CH); 48.60 (CH_2_); 38.57 (CH_2_); 38.46 (CH_2_); 38.10 (CH_2_); 35.78 (CH_2_); 35.10 (CH_2_); 34.11 (CH_2_); 31.35 (CH_2_); 30.78 (CH_2_); 28.28 (CH_2_); 25.01 (CH_2_); 22.82 (CH_3_). ^195^Pt NMR [DMSO-d_6_, δ (ppm)]: 1219.

##### Synthesis of TA-PEG_6_-COOH

40 mg (0.11 mmol) of **NH_2_-PEG_6_-COOH** were dissolved in 5 mL of dried DMF and 30 µL (0.17 mmol) of DIPEA were added, followed by 39.4 mg (0.13 mmol) of TA-NHS. The mixture was stirred overnight at room temperature under N_2_. The solvent was evaporated under low pressure and the compound purified by HPLC. The collected fractions were freeze-dried, after removal of the organic solvent under vacuum, to afford **TA-PEG_6_-COOH** (42.8 mg, 0.08 mmol) (Ƞ = 71.8%). ESI-MS (CH_3_CN): m/z: [C_23_H_43_NO_9_S_2_H]^+^: calculated = 542.2; found = 542.4. [C_23_H_43_NO_9_S_2_Na]^+^: calculated = 564.2; found = 564.3. [C_23_H_43_NO_9_S_2_K]^+^: calculated = 580.2; found = 580.2. ^1^H NMR [CD_3_OD, δ (ppm)]: 3.76 (t, 2H, CH_2_); 3.64 (m, 18H, 9CH_2_ & 1H, CH); 3.55 (t, 2H, CH_2_); 3.36 (m, 2H, CH_2_); 3.05-3.24 (m, 2H, CH_2_); 2.56 (m, 2H, CH_2_); 2.46 (m, 1H, CH_2_); 2.23 (t, 2H, CH_2_); 1.91 (m, 1H, CH_2_); 155-180 (m, 4H, CH_2_); 1.46 (m, 2H, CH_2_). ^13^C NMR [CD_3_OD, δ (ppm)]: 175.96 (C=O), 175.10 (C=O); 71.41 (8CH_2_); 71.20 (CH_2_); 70.51 (CH_2_); 67.68 (CH_2_); 67.57 (CH_2_); 57.60 (CH); 41.55 (CH_2_); 40.42 (CH_2_); 39.13 (CH_2_); 36.65 (CH_2_); 35.54 (CH_2_); 29.34 (CH_2_); 26.65 (CH_2_).

##### Synthesis of TA-Pt3

12.6 mg of **Pt-Ala** (0.028 mmol) were dissolved in 2 mL of dried DMF and 10 µL (0.057 mmol mmol) of DIPEA were added. Thereafter, 42.8 mg (0.028 mmol) of **TA-PEG6-COOH** were dissolved in 2 mL of dried DMF and 11 mg (0.028 mmol) of HBTU were added. Then, the two solutions were combined, and the mixture was stirred overnight at room temperature under N_2_. Then, the compound was purified by HPLC. The collected fractions were freeze-dried, after removal of the organic solvent under vacuum, to afford **TA-Pt3** (3.9 mg, 0.003 mmol) (Ƞ = 14.3%).

R_T_ (HPLC system 2): 25.34 min. ESI-MS (CH_3_OH): m/z: [C_28_H_56_C_l2_N_4_O_12_PtS_2_Na]^+^: calculated = 993.2; found = 993.4. ^1^H NMR [CD_3_OD, δ (ppm)]: 3.75 (t, 2H, CH_2_); 3.65 (m, 20H, 10CH_2_); 3.55 (t, 2H, CH_2_); 3.45-3.37 (br, 6H, 3CH_2_); 3.14 (m, 2H, CH_2_); 2.57-2.40 (br, 4H, 2CH_2_ & 1H, CH); 2.22 (t, 2H, CH_2_); 2.06 (s, 3H, CH_3_); 1.90 (m, 2H, CH_2_); 1.77-1.57 (br, 6H, 3CH_2_; 1.48 (m, 2H, CH_2_). ^13^C NMR [CD_3_OD, δ (ppm)]: 181.33 (C=O); 175.83 (C=O); 173.76 (2 C=O); 71.20 (8CH_2_); 70.99 (CH_2_); 70.96 (CH_2_); 70.33 (CH_2_); 67.91 (CH_2_); 57.32 (CH); 41.06 (CH_2_); 40.08 (CH_2_); 39.08 (CH_2_); 37.40 (CH_2_); 37.13 (CH_2_); 37.00 (CH_2_); 36.56(CH_2_); 35.40 (CH_2_); 29.60 (CH_2_); 26.45 (CH_2_); 22.37 (CH_3_). ^195^Pt NMR [CD_3_OD, δ (ppm)]: 1087.

### Synthesis and Characterization of Pt(IV) Prodrug-Containing AuNPs

#### Synthesis of AuNP-BBN-Pt1

140 µL of DMSO were added to 160 µL of **AuNP-BBN** (5 mg/mL in DI water), followed by 1.6 mg (0.0023 mmol) of **TA-Pt1** in 300 µL of DMSO. The mixture was stirred overnight at room temperature. The solution was centrifuged at 12000 rpm for 5 min and the nanoconjugates were washed H_2_O and lyophilized.

#### Synthesis of AuNP-BBN-Pt2

140 µL of DMSO were added to 160 µL of **AuNP-BBN** (5 mg/mL in DI water), followed by 1.6 mg (0.002 mmol) of **TA-Pt2** in 160 µL of DMSO. The mixture was stirred overnight at room temperature. The solution was centrifuged at 12000 rpm for 5 min and the nanoconjugates were washed H_2_O and lyophilized.

#### Synthesis of AuNP-BBN-Pt3

130 µL of CH_3_OH were added to 130 µL of **Au-BBN** (5 mg/mL in DI water), followed by 1.3 mg (0.0013 mmol) of TA-PEG6-Pt in 130 µL of CH_3_OH. The mixture was stirred overnight at room temperature. The solution was centrifuged at 12000 rpm for 5 min and the nanoconjugates were washed with CH_3_OH and H_2_O and lyophilized.

#### Characterization of the AuNPs

##### Dynamic Light Scattering (DLS) and Zeta Potential Determination

DLS measurements were performed with a Malvern Zetasizer Nano ZS (Malvern Instruments Ltd., Worcestershire, UK) equipped with a 633 nm He-Ne laser and operating at an angle of 173°. The software used to collect and analyze the data was the Dispersion Technology Software (DTS) version 5.10 from Malvern. 600 μL of each sample was measured in low volume semi-micro disposable sizing cuvettes (Fisher Scientific, USA) with a path length of 10 mm. Triplicate measurements were made at a position of 4.65 mm from the cuvette wall with an automatic attenuator. For each sample, 15 runs of 10 s were performed. The size distribution, the Z-average diameter and the polydispersity index (PDI) were obtained from the autocorrelation function using the “general purpose mode” for all nanoparticle samples. The default filter factor of 50% and the default lower threshold of 0.05 and upper threshold of 0.01 were used. Zeta potential measurements were performed in triplicates using water as a dispersant and the Huckel model. For each sample, 20 runs were performed in autoanalysis mode.

##### Transmission Electron Microscopy (TEM) Transmission

Electron microscope images were obtained on a JEOL 1400 transmission electron microscope (TEM, JEOL LTD., Tokyo, Japan). TEM samples were prepared by placing 5 μL of gold nanoparticle solution on the 300-mesh carbon coated copper grid. Excess solution was removed carefully, and the grid was allowed to dry for an additional five minutes. The average size and size distribution of the nanoparticles were determined by processing the TEM image Adobe Photoshop with Fovea plug-ins.

##### Determination of Platinum content by inductively coupled plasma mass (ICP-MS)

Samples were dissolved in 0.5 mL nitric acid (65%) with a hot plate at 100 °C during 12 h. The solution digest was diluted in ultrapure water to 10 mL, obtained from a MilliQ apparatus. The Pt content was measured by a Thermo X-Series Quadrupole ICP-MS (Thermo Scientific) equipped with Ni cones and a glass concentric nebulizer (Meinhard, 1.0 mL min^-1^) refrigerated with a Peltier system. Indium (^115^In) at a concentration of 10 μg L^-1^ was used as internal standard. Standards were prepared from standard ICP-MS 71 A (Inorganic Venture) with a final concentration of 5.0% nitric acid.

### Radiochemistry

#### Labeling with ^67^Ga

In a 2 mL eppendorf, 350 µL of 0.4 M ammonium acetate (pH ≈ 7) were mixed with 30 µL of **AuNP-BBN-Pt1** (5 mg/mL in DI water) or 40 µL of **AuNP-BBN-Pt2**/**AuNP-BBN-Pt3** (5 mg/mL in DI water). To this mixture, 250 µL of ^67^GaCl_3_ in 0.1 M HCl (20-70 MBq) were added. The mixture was heated at 70 °C for 30 minutes The solution was then filtered in a Millipore Amicon Ultra 0.5 mL 10k to recover the ^67^Ga-labeled AuNPs (radiochemical yield > 95 %). The radiochemical purity of the collected gold nanoconstructs was assessed by radio-TLC (> 95 %), using an ITLC-SG support and CH_3_OH/HCl 6M (95:5) as eluent system.

#### *In vitro* Stability Studies

The radiochemical stability of the radiolabeled nanoparticles was assessed in cell culture medium (DMEM with 1% FBS (fetal bovine serum) and 1% antibiotics), PBS 0.1 M, and in the presence of apo-transferrin (3 mg/mL in 10 mM HNaCO_3_). To the radiolabeled AuNPs (40 μL solution) were added 160 μL of the different challenging solutions and the resulting mixtures were incubated at 37 °C for different periods of time (0 to 24 h). For each time point, the mixtures were analyzed by radio-TLC using an ITLC-SG support and CH_3_OH/HCl 6M (95:5) as eluent system.

### Cellular Studies

PC3 (androgen independent prostate cancer cells, ATCC) and RWPE-1 (non-neoplastic androgen responsive prostate epithelial cells, ATCC) were cultured in RPMI supplemented with FBS (10%) and keratinocyte growth medium supplemented with bovine pituitary extract (BPE, 0.05 mg/ml) and human recombinant epidermal growth factor (EGF, 5 ng/ml), respectively.

#### Receptor binding affinity studies

The binding affinity of the **AuNP-BBN-Pt** (Pt = Pt1-Pt3) towards the GRPR was determined by a competitive binding assay on the prostate carcinoma cell line PC3 using [^125^I-Tyr4]BBN (PerkinElmer, Inc., Boston, MA, USA) as the GRPR-specific radioligand. Briefly, cells were seeded in 24-well plates (150,000 cells per well) and allowed to attach overnight. On the day of the experiment, cells were rinsed with ice-cold binding assay medium (RPMI 1640 medium supplemented with 1% (v/v) FBS, 25 mM HEPES and 1% (w/v) BSA). Competition was conducted by incubation of 20,000 cpm of [^125^I-Tyr4]BBN (0.2 mL) in the presence of increasing concentrations of **AuNP-BBN-Pt** in 0.1 mL of the binding buffer (total volume per well 0.3 mL) such as the final concentrations ranged from 0.001-100 µg/mL, in triplicate. The incubation was done for 90 min at 4 °C. The competition was interrupted by discarding supernatants and washing the cells twice with ice-cold PBS with 0.2% BSA. Cells were then lysed with 1 M NaOH treatment (2 × 0.4 mL, 10 min at 37 °C). Lysates were collected and counted for their radioactivity content in an automated γ-counter (HIDEX AMG, Hidex, Turku, Finland). The percentage of [^125^I-Tyr4]BBN bound to cells was plotted against increasing concentrations of the **AuNP-BBN-Pt**. The IC_50_ values (concentration of competitor required to inhibit 50% of the radioligand binding) were calculated by nonlinear regression according to a one-site model using GraphPad PRISM 9 graphing software (San Diego, CA, USA).

#### Cellular uptake and internalization studies by radiometric measurements

Cellular uptake and internalization assays of **^67^Ga-AuNP-BBN-Pt** (Pt = Pt1-Pt3) were performed in quadruplicate in PC3 and RWPE-1 cells seeded at a density of 2 x 10^5^ cells in 0.5 mL of culture medium per well in 24 well-plates and allowed to attach overnight. The cells were incubated at 37 ºC for a period of 15 min to 3 h with about 400,000 cpm of the radiolabeled **AuNP-BBN-Pt** in 0.5 mL of culture medium (RPMI, 10% FBS, 1% Penicilin/streptomycin). Incubation was terminated by washing the cells with ice-cold medium. Cell surface-bound radioactivity was removed by two steps of acid wash (50 mM glycine, HCl/100 mM NaCl, pH 2.8) at room temperature for 4 min. The pH was neutralized with cold PBS, and subsequently the cells were lysed by 10 min incubation with 1 M NaOH at 37 ºC to determine internalized nanoparticles. The radioactivity associated to each fraction in the cells was measured in gamma counter and expressed as the percentage of the total activity added to the cells and presented as an average plus the standard deviation.

#### Cellular uptake studies by PIXE analysis

The concentrations of Au and Pt in PC3 cells, incubated with **AuNP-BBN-Pt1** at 50 µM (in terms of Pt) for 24 h, were determined by particle-induced X-ray emission (PIXE) technique using the Van de Graaff accelerator installed at Instituto Superior Técnico, Campus Tecnológico e Nuclear. The cell pellets were obtained by centrifugation after washing the cells with PBS to remove the culture medium, freeze-dried and digested using suprapure reagents, nitric and hydrochloric acids (1:3 molar ratio) together with yttrium (Y) (100 mg/l) as an internal standard. The procedure combined ultrasound cycles of 30 min at 60 °C and microwave-assisted acid digestion (350 W, 15 s). The detailed methodology encompassing PIXE analysis, concentration calculation and quality control, was previously described 50, 51. The elemental concentrations of Au and Pt were obtained in µg/g and converted to ng/10^6^ cells.

#### Cytotoxicity Assays

Cells were seeded in 96-well plates (1-5 x10^4^ cells/200 µL medium) and incubated at 37 °C for 24 h to adhere. Then, the medium was discarded, and cells were incubated with the desired AuNPs and cisplatin at serial dilutions in the range 0.1-100 µM (calculated as Pt concentration) for 72 h. After incubation, the viability was determined using the MTT assay. For the assay, the culture medium was removed and 200 μL of MTT solution (0.5 mg/mL) was added to each well. After incubation for 3 h at 37°C, the MTT solution was discarded and 200 μL of DMSO was added to dissolve the formazan crystals. The absorbance was measured at 570 nm using an ELISA plate reader. Absorbance readings from treated samples were normalized to controls and dose response curves to calculate the IC_50_ values were obtained using the Graph Pad Prism 5 software.

#### ROS Assay

The detection of intracellular ROS levels, mainly H_2_O_2_, in the PC3 cells was done using the molecular probe 2',7'-dichlorofluorescein diacetate (H_2_DCF-DA) 52, 53. This cell-permeant probe upon cleavage of the acetate groups within the cells by intracellular esterases is converted to highly fluorescent 2',7'-dichlorofluorescin (DCF) upon oxidation with ROS. For the assays, cells (2×10^4^ cells/well) were seeded in 96-well plates and left to adhere overnight. The medium was then replaced with a solution of 10 μM H_2_DCF-DA in colorless DMEM (FluoroBrite™ DMEM, Gibco) and cells were incubated at 37 °C for 30 min. Then, the medium was aspirated, the cells washed with PBS and cells were then incubated with the AuNPs in fresh medium at selected concentrations 10, 20 and 50 µM (in terms of Pt) for 1 h. DCF fluorescence was measured using a Varioskan Lux multimode microplate reader (Thermo Fisher Scientific) at 492 nm excitation and 517 nm emission. Results of fluorescence were expressed as the fold change in fluorescence levels compared with controls.

#### Apoptosis Assay

The activities of caspase-3 and caspase-7, both involved in apoptosis, were measured using the luminescent Caspase-Glo® 3/7 assay kit (Promega) 53. The assay provides a luminogenic caspase-3/7 substrate containing the tetrapeptide sequence Asp-Glu-Val-Asp (DEVD aminoluciferin) in a reagent optimized for caspase activity, luciferase activity and cell lysis. In the presence of caspase 3/7 this substrate is cleaved, aminoluciferin is released, consumed by luciferase (provided in the reaction mixture), which generates a luminescent signal which is proportional to the amount of caspase activity present in the cells. The assay was carried out in PC3 cells, plated in 96 wells, treated with **AuNP-BBN-Pt1** or cisplatin at 20 and 50 µM (in terms of Pt) for 48 and 72h. After incubation, 100 μL of medium was removed from each well, Caspase 3/7® reagent was added in a 1:1 ratio and the plate was shaken in an orbital shaker for 30 s at 300-500 rpm. The plate was incubated at room temperature and protected from light for 1.5 h. The luminescence intensity was measured using a Varioskan LUX scanning multimode reader (Thermo Fisher Scientific). Each concentration was tested with at least three replicates.

### Biodistribution and Small Animal Imaging Studies

Animal experiments were performed in compliance with national and European legislation for Animal Care and Ethics in Animal Experimentation. The animals were housed in a temperature- and humidity-controlled room with a 12 h light/12 h dark schedule.

#### Biodistribution

Biodistribution and the ability of **^67^Ga-AuNP-BBN-Pt1** to be retained in tumors was evaluated in 10-12 weeks old Balb/c-Nude mice (obtained from Charles River, Spain) with PC3 xenografts, weighing approximately 15-19 g. A 150 μL bolus containing a suspension of approximately 8 X10^6^ freshly harvested human PC3 cells in Matrigel:PBS buffer 1:1 was subcutaneously injected in the right flank of each female nu/nu mouse. The animals were kept under aseptic conditions and approximately 10 days later developed well-palpable tumors at the inoculation site.

Xenograft-bearing animals were intratumorally injected with the nanoparticle suspension (1.4-4.6 MBq) in 50 μL of NaCl 0.9%. The injection was given at 2-3 different spots in each tumor. Mice were sacrificed in groups of 3, by cervical dislocation at 1 h, 24 h and 72 h after injection. The dose administered and the radioactivity in the sacrificed animals was measured using a dose calibrator (Capintec CRC25R). The difference between the radioactivity in the injected and sacrificed animals was assumed to be due to excretion. Tissues of interest were dissected, rinsed to remove excess blood, weighed, and their radioactivity was measured using a gamma counter (HIDEX AMG, Hidex, Turku, Finland). The uptake in the tissues was calculated and expressed as a percentage of the total injected activity per organ (% I.D.) and as a percentage of the injected activity per gram of tissue (% I.D./g). For blood, bone, muscle, total activity was estimated assuming that these organs constitute 6, 10, 40% of the total body weight, respectively. After complete decay of ^67^Ga, tumors were also analyzed by ICP-MS to assess the Pt and Au content, expressed as a percentage of the total injected dose per tumor (% I.D.). For that, the Pt and Au amount in the injected solution was also determined. The amount of Pt content accumulated into the tumor was also analyzed in the same animal model after injection of a cisplatin solution with approximately the same Pt concentration (0.63 µg/50 µL), at the same time points.

#### MicroSPECT/CT imaging

SPECT/CT imaging studies were also conducted in male Balb/c Nude mice bearing PC3 tumor xenografts at 1, 24 and 72 hours after intratumoral injection of **^67^Ga-AuNP-BBN-Pt1** (2x50 μL, 10 MBq). Prior to the image acquisitions, the mice were anesthetized with isofluorane and positioned on the animal bed.

To perform the acquisitions, a FLEX® Triumph® II (Trifoil Imaging) SPECT/CT imaging system was used. The system was equipped with one Cadmium Zinc Telluride (CZT) detector and a five-pinhole (1.0 mm) collimator (N5F75A10). The SPECT data acquisitions were performed at 60 seconds per projection, with the stepwise rotation of 64 projections over 360º, accounting for the decay of ^67^Ga at 24 h and 72 h p.i. (84 s and 96 s per projection, respectively). Cone-beam CT images were acquired in a gantry fly motion mode (512 projections, 110 ms/projection, 70 kVp, 340 μA), 2X2 binning, 1184 x 1120 matrix. SPECT Triumph Reconstruction Application v1.0.8.0 (Trifoil Inc.) was used to obtain the transaxial, coronal, and sagittal slices, using a 3D-ordered subset expectation maximization (3D-OSEM) reconstruction algorithm with 8 subsets and 5 iterations.

Vivid Amira 4.1 software was used to perform the post-processing analysis, as well as the co-registration of SPECT images in conjunction with the corresponding CT images. A gauss filter with a 0.6 sigma, 3x3 kernel, was applied to the CT slices at a threshold of 1300.
