# Supplementary material for: Image-Guided Nanodelivery of Pt(IV) Prodrugs to GRP-Receptor Positive Tumors
Source: Nanotheranostics. 2023 Jan 1;7(1):22–40. doi: 10.7150/ntno.78807 (PMC9760368; doi:10.7150/ntno.78807)
Supplement: Supplementary file 1 — Supplementary figures and table. [file ntnov07p0022s1.pdf]

Supplementary Information

Image-Guided Nanodelivery of Pt(IV) Prodrugs to GRP-Receptor Positive Tumors

Francisco Silva[1,*], Carolina Mendes[1], Alice D'Onofrio[1], Maria Paula Cabral Campello[1,2], Fernanda Marques[1,2], Teresa Pinheiro[2,3], Kyle Gonçalves[1], Sérgio Figueiredo[4,5], Lurdes Gano[1,2], Mauro Ravera[6], António Paulo[1,2*]

Index

Figure S 1. Schematic synthesis of the pegylated thioctic acid precursors. **1**

Figure S 2. ESI-MS spectra. **1**

Figure S 3. ^{1}H, ^{13}C and ^{195}Pt NMR spectra in DMSO-d_6 of **AuNP-BBN-Pt1**. **2**

Figure S 4. ^{1}H, ^{13}C and ^{195}Pt NMR spectra in DMSO-d_6 of **AuNP-BBN-Pt**2. **3**

Figure S 5. TEM imaging of the Pt(IV) prodrug-containing AuNPs. **4**

Figure S 6. iTLC radiochromatograms of **^{67}Ga-AuNP-BBN-Pt2** and **^{67}Ga-AuNP-BBN-Pt3**. **4**

Figure S 7. Binding Affinity curves and IC_{50} values of the Pt(IV) prodrug-containing AuNPs. **5**

Table S 1. Biodistribution study of **^{67}Ga-AuNP-BBN-Pt1** in PC3 xenograft model, upon bolus intratumoral administration performed at 1, 24 and 72 h post-administration. **6**

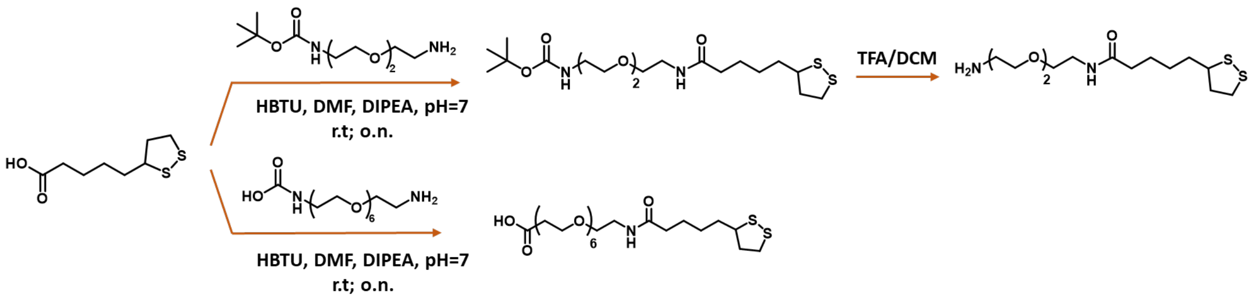

Figure S 1. Schematic synthesis of the pegylated thioctic acid precursors.

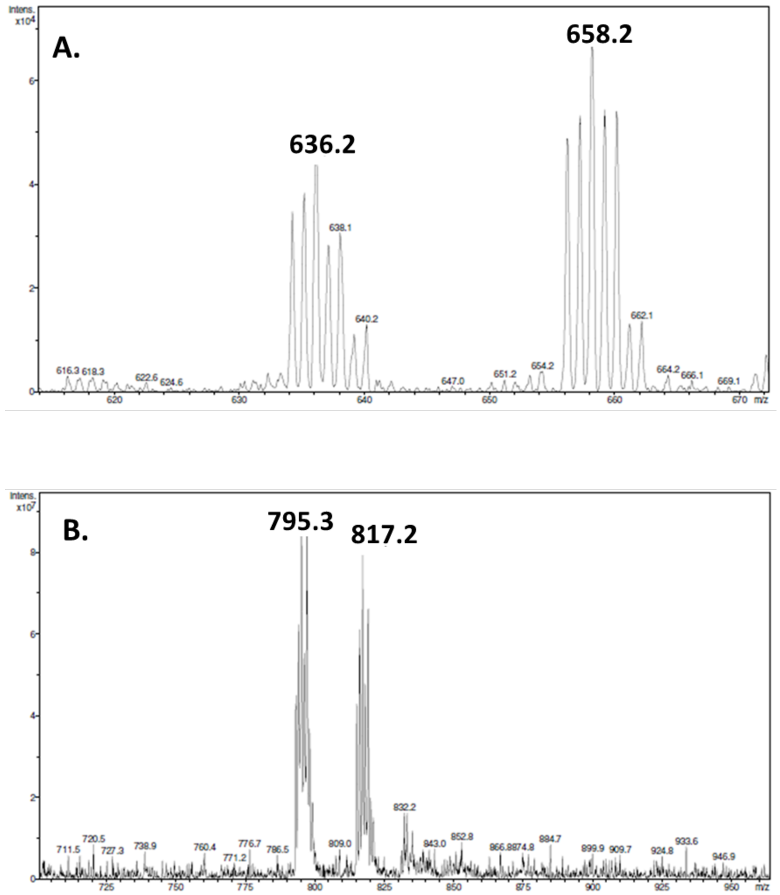

Figure S 2. A. TA-Pt1: m/z $[C_{13}H_{27}Cl_2N_3O_5PtS_2H]^+$: calc. = 636.0; found = 636.2, $[C_{13}H_{27}Cl_2N_3O_5PtS_2\text{-}Na]^+$: calc. = 658.0; found = 658.2. **B. TA-Pt2**: m/z: $[C_{20}H_{40}PtCl_2N_4O_8S_2H]^+$ calc. = 795.1; found = 795.3 $[C_{20}H_{40}PtCl_2N_4O_8S_2Na]^+$ calc. = 817.1; found = 817.2.

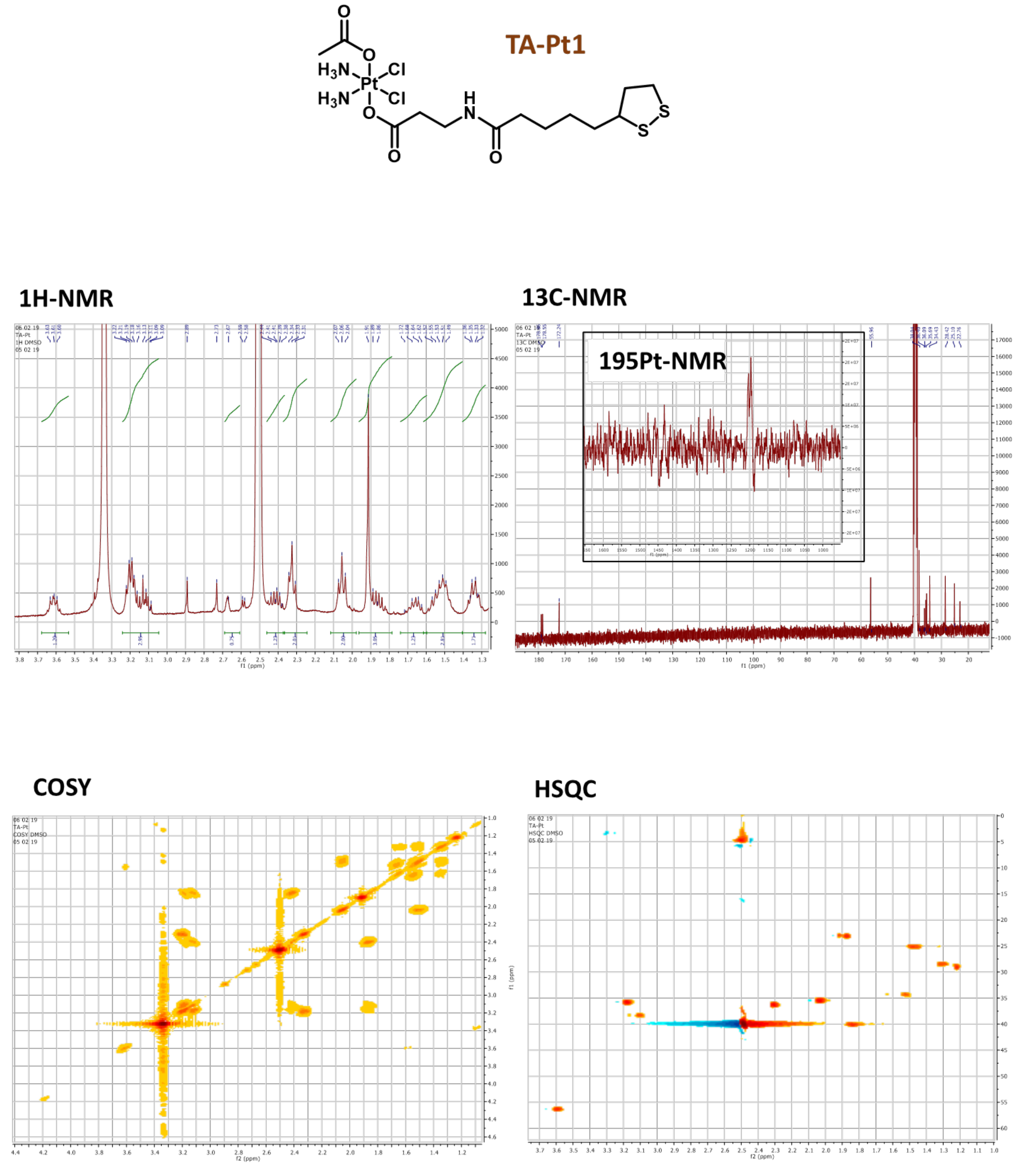

Figure S 3. Characterization of the thioctic acid-containing Pt(IV) prodrug **TA-Pt1** by multinuclear ^{1}H, ^{13}C and ^{195}Pt NMR analysis in DMSO-d_6.

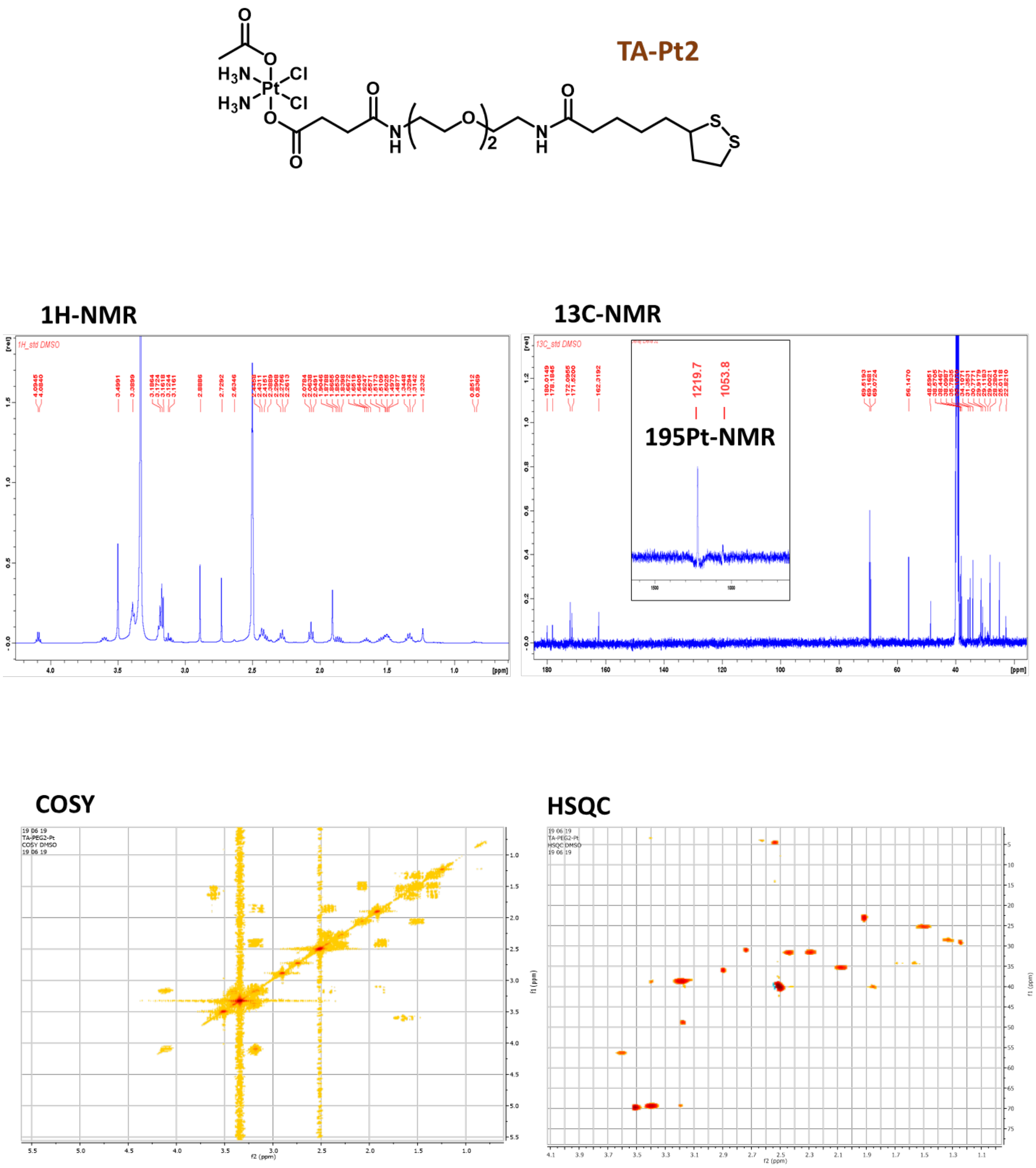

Figure S 4. Characterization of theathioctic acid-containing Pt(IV) prodrug **TA-Pt2** by multinuclear ^{1}H, ^{13}C and ^{195}Pt NMR analysis in DMSO-d_6.

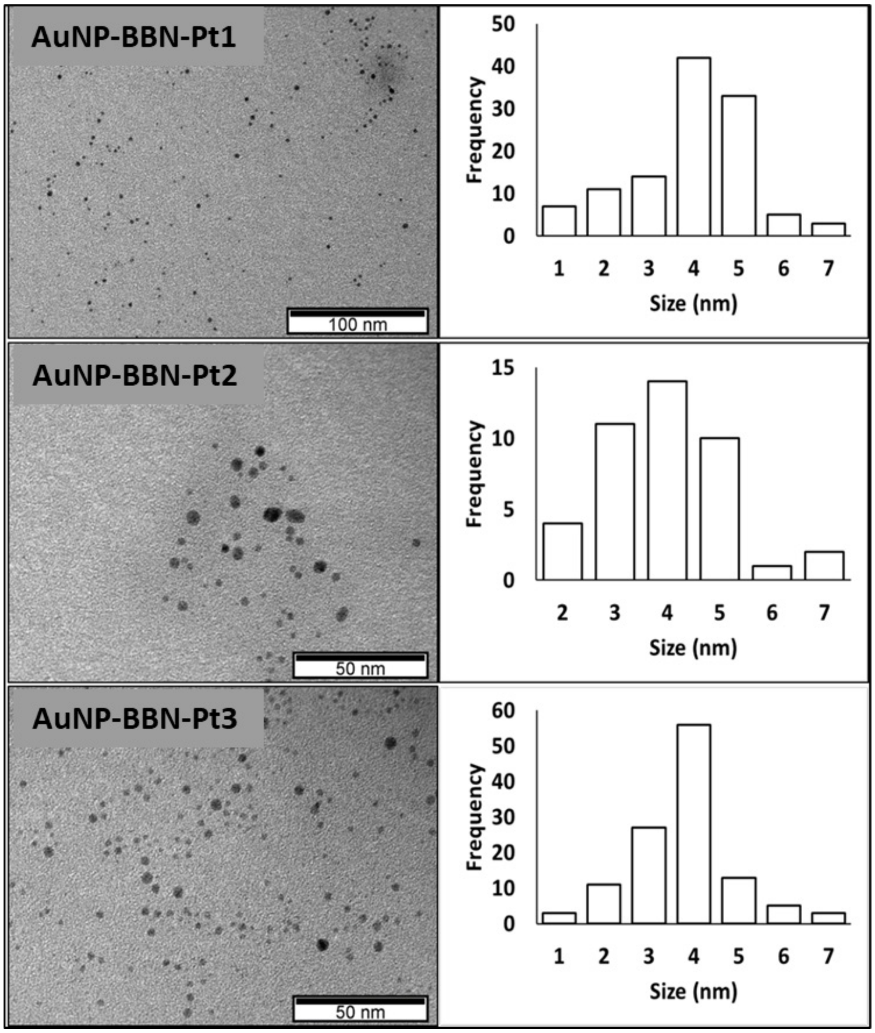

Figure S 5. TEM imaging of the Pt(IV) prodrug-containing AuNPs.

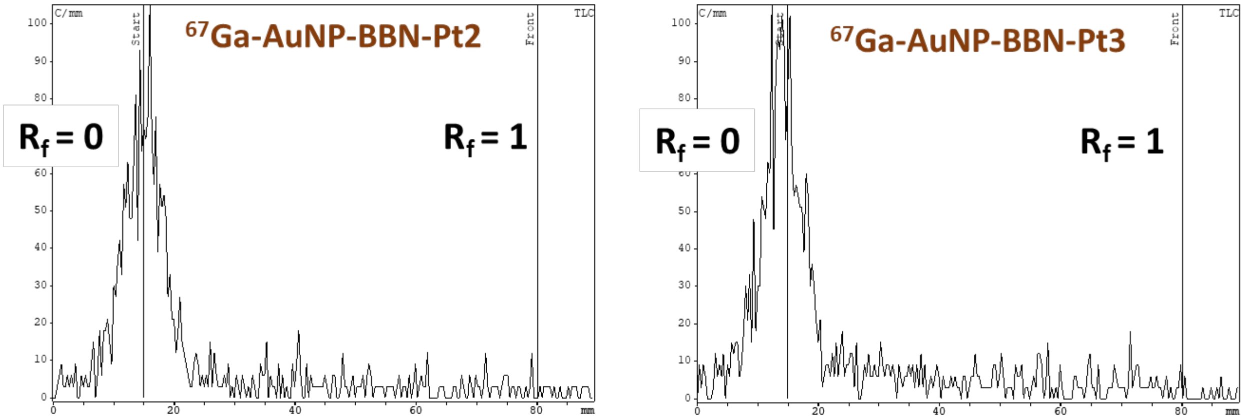

Figure S 6. iTLC radiochromatograms of ^{67}**Ga-AuNP-BBN-Pt2** and ^{67}**Ga-AuNP-BBN-Pt3**.

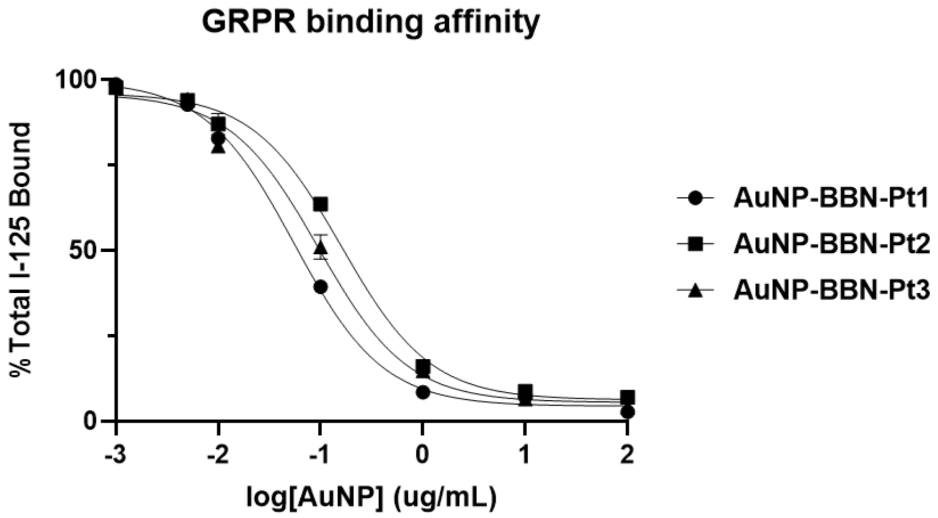

Compound	IC_{50}
AuNP-BBN-Pt1	0.055 ± 0.005 µg/mL
AuNP-BBN-Pt2	0.160 ± 0.022 µg/mL
AuNP-BBN-Pt3	0.096 ± 0.019 µg/mL

Figure S 7. Binding Affinity curves and IC_{50} values of the Pt(IV) prodrug-containing AuNPs obtained by competitive binding assay using PC3 cells and [^{125}I-Tyr4]BBN as the GRPR-specific radioligand.

Table S 1. Biodistribution data of ^{67}**Ga-AuNP-BBN-Pt1** in PC3 xenograft model, upon bolus intratumoral administration, performed at 1, 24 and 72 h post-administration.

**Organ**	**% Injected Dose/organ**		
	**1 h**	**24 h**	**72 h**
**Blood**	3.4 ± 0.7	1.29 ± 0.02	0.43 ± 0.08
**Liver**	0.7 ± 0.3	7.0 ± 0.9	4.7 ± 0.6
**Intestine**	0.9 ± 0.4	3.1 ± 0.2	2.3 ± 0.2
**Spleen**	0.04 ± 0.01	0.43 ± 0.01	0.17 ± 0.02
**Heart**	0.14 ± 0.01	0.05 ± 0.01	0.06 ± 0.01
**Lung**	0.16 ± 0.07	1.1 ± 0.2	0.20 ± 0.07
**Kidney**	0.3 ± 0.1	1.1 ± 0.1	1.3 ± 0.1
**Muscle**	2.3 ± 1.0	2.2 ± 0.1	2.5 ± 0.3
**Bone**	0.9 ± 0.3	4.6 ± 0.7	8.3 ± 1.0
**Stomach**	0.13 ± 0.05	0.28 ± 0.03	0.25 ± 0.02
**Pancreas**	0.08 ± 0.02	0.19 ± 0.06	0.22 ± 0.09
**Brain**	0.03 ± 0.01	0.05 ± 0.01	0.04 ± 0.01
**Tumor**	77.5 ± 0.7	34.3 ± 1.6	25.6 ± 10.6
**Excretion**	2.2 ± 1.0	34.5 ± 0.8	40.7 ± 2.6
